# The *App-Runx1* Region Is Critical for Birth Defects and Electrocardiographic Dysfunctions Observed in a Down Syndrome Mouse Model

**DOI:** 10.1371/journal.pgen.1002724

**Published:** 2012-05-31

**Authors:** Matthieu Raveau, Jacques M. Lignon, Valérie Nalesso, Arnaud Duchon, Yoram Groner, Andrew J. Sharp, Doulaye Dembele, Véronique Brault, Yann Hérault

**Affiliations:** 1 Institut de Génétique et de Biologie Moléculaire et Cellulaire, Department of Translational Medicine and Neurogenetics, CNRS, INSERM, Université de Strasbourg, UMR7104, UMR964, Illkirch, France; 2 Immunologie et Embryologie Moléculaire, CNRS Université d'Orléans, UMR6218, Orléans, France; 3 Department of Molecular Genetics, The Weizmann Institute of Science, Rehovot, Israel; 4 Department of Genetics and Genomic Sciences, Mount Sinai School of Medicine, New York, New York, United States of America; 5 Transgénèse et Archivage d'Animaux Modèles, CNRS, UPS44, Orléans, France; 6 Institut Clinique de la Souris, Illkirch, France; University College London, United Kingdom

## Abstract

Down syndrome (DS) leads to complex phenotypes and is the main genetic cause of birth defects and heart diseases. The Ts65Dn DS mouse model is trisomic for the distal part of mouse chromosome 16 and displays similar features with post-natal lethality and cardiovascular defects. In order to better understand these defects, we defined electrocardiogram (ECG) with a precordial set-up, and we found conduction defects and modifications in wave shape, amplitudes, and durations in Ts65Dn mice. By using a genetic approach consisting of crossing Ts65Dn mice with Ms5Yah mice monosomic for the *App*-*Runx1* genetic interval, we showed that the Ts65Dn viability and ECG were improved by this reduction of gene copy number. Whole-genome expression studies confirmed gene dosage effect in Ts65Dn, Ms5Yah, and Ts65Dn/Ms5Yah hearts and showed an overall perturbation of pathways connected to post-natal lethality (*Coq7, Dyrk1a, F5, Gabpa, Hmgn1, Pde10a, Morc3, Slc5a3*, and *Vwf*) and heart function (*Tfb1m, Adam19, Slc8a1/Ncx1*, and *Rcan1*). In addition cardiac connexins (*Cx40, Cx43*) and sodium channel sub-units (*Scn5a, Scn1b, Scn10a*) were found down-regulated in Ts65Dn atria with additional down-regulation of *Cx40* in Ts65Dn ventricles and were likely contributing to conduction defects. All these data pinpoint new cardiac phenotypes in the Ts65Dn, mimicking aspects of human DS features and pathways altered in the mouse model. In addition they highlight the role of the *App*-*Runx1* interval, including *Sod1* and *Tiam1*, in the induction of post-natal lethality and of the cardiac conduction defects in Ts65Dn. These results might lead to new therapeutic strategies to improve the care of DS people.

## Author Summary

Down syndrome is a common cause of birth defects mainly due to heart diseases with an incidence of up to 50%. Nevertheless the pathophysiology of DS cardiac anomalies and complications are not well understood. Here we have been able to demonstrate using a series of DS mouse models that birth defects and changes in ECG, similar to those in humans, are linked to dosage sensitive genes located in the *App-Runx1* genetic interval. Further molecular characterization revealed an overall perturbation of transcription, a few candidates homologous to human chromosome 21 genes, and changes in the expression of several genes, including cardiac connexins (*Cx40, Cx43*) and sodium channel sub-units (*Scn5a, Scn1b, Scn10a*), likely contributing to ECG defects. This model provides a unique opportunity to study further the DS heart diseases and to propose therapeutic avenues for treatment of DS cardiac complications observed in adults.

## Introduction

Down syndrome (DS), caused by trisomy of human chromosome 21 (Hsa21; Hsa for *Homo sapiens*) is the most common chromosomal anomaly and cause of intellectual disabilities [1]–[4]. Among DS newborns, 40% to 60% are affected by congenital heart defects (CHD), consisting mainly of atrioventricular septal defects (AVSD) with additional changes in the cardiac axis and ECG [5], [6]. In comparison incidence of CHD in all newborns is between 8‰ and 14‰ and is the leading cause of death before 1 year of age [5]. Thanks to cardiac surgery and medical care, CHD patient's life expectancy has increased from 12 years in the 1940s to 60 years nowadays [1], [5]. However, even in the absence of overt CHD, a number of functional anomalies have been observed in adult DS patients, such as altered heart rate regulation [7], [8], valvular dysfunction [9], [10], bradycardia and AV block [11], [12]. Correspondingly, adult DS population has increased and requires prolonged follow up and care, particularly in the cardiovascular field.

To further study the correlation between phenotype and genotype in DS, various mouse models have been created [2], [4]. Indeed, the long arm of Hsa21 is approximately 33.9 Mb in length and contains about 430 protein coding genes (either known or putative) of which 293 have a homolog in the mouse genome according to the NCBI37/mm9 genome sequence. Among those, about 235 genes are located on syntenic regions on mouse chromosomes 16 (Mmu for *Mus musculus*; Mmu16, 23.3 Mb, 166 genes), 17 (Mmu17, 1.1 Mb, 22 genes) and 10 (Mmu10, 2.3 Mb, 47 genes). The largest duplication created on the Mmu16, the Dp(16)1Yey, results in a mouse trisomic for the whole region from *Lipi* to *Zfp295* that shows cardiac anomalies [13]. Similarly the Ts(17^16^)65Dn (Ts65Dn) model, which carries a shorter trisomy ranging from *Mrpl39* to *Zfp295*, shows specific cardiovascular malformations associated with post-natal lethality and reduced transmission rate of the Ts65Dn allele at weaning [14], [15]. This is also observed in trisomy for the *Tiam1-Kcnj6* region [16]. Thus candidate genes for cardiac and lethality phenotypes should be located on the Hsa21 homologous region present on Mmu16.

At present, while morphological and histological aspects of the cardiovascular phenotypes observed in DS mouse models during perinatal age have been described, the functional aspects remain unexplored in viable adult animals. Before the development of cardiac imaging, ASD, VSD and AVSD were scored by abnormal electrocardiographic recordings (ECG) in man. CHD could be predicted on this basis in 80% of the cases in DS people. Most characteristic features of CHD-induced changes in ECG are superior frontal QRS axis deviation, first degree block and partial bundle branch block [6], [17]–[19]. QRS prolongation and P wave axis changes are also common features [17]. When combined to clinical examination and radiography, ECG becomes highly sensitive and specific even though the best investigation remains echocardiography.

The aim of the present study was to explore the cardiac function in the Ts65Dn mouse model. Moreover we wanted to assess the susceptibility to dosage effect of genes within the *App* to *Runx1* region and their implications in heart defects and lethality by a subtractive approach. For that we used a partial monosomic model, Ms5Yah, carrying a deletion for the previously mentioned region and we found that compound Ts65Dn/Ms5Yah mice were partially rescued for the early post-natal lethality and some aspect of ECG features. Furthermore re-establishing euploidy in the *App-Runx1* region modified gene expression changes, highlighting several pathways involved in cardiac function that were deregulated in the Ts65Dn DS mouse model.

## Results

### Ts65Dn transmission rate and heart anatomy and histology

**Figure 1:**
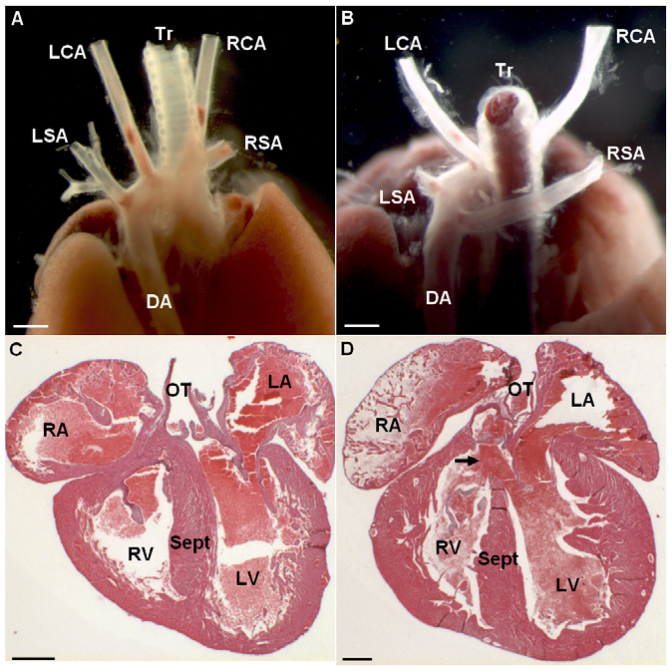
Changes in cardiovascular morphology observed in the Ts65Dn model. Posterior view of a wt individual illustrates the normal location of the aorta and efferent vessels (A). Posterior view of a Ts65Dn individual shows an aberrant right subclavian artery (RSA) arising from the distal part of the aortic arch and describing a retro-oesophagian loop (B). Haematoxylin-eosin staining of histological cross-sections illustrates full septation of the ventricles with individualized valves in wt (C) while Ts65Dn heart cross-section shows an upper communication of the ventricles (D; arrow). This cardiovascular anomaly was observed in 1 in 18 Ts65Dn dead newborns. LSA, left subclavian artery; RSA, right subclavian artery; DA, descending aorta; RA, right atrium; LA, left atrium; RV, right ventricle; LV, left ventricle; Sept, septum; OT, outflow tract. Scale bars represent 1 mm in A, B and 400 µm in C,

As a preliminary experiment, transmission rate of the Ts65Dn allele at weaning was evaluated on a B6C3B mixed strain. Among 617 mice born from Ts65Dn x B6C3B crosses, 34% Ts65Dn individuals were observed at weaning (Table 1), far below the 50% that could be expected (χ^2^ *P* = 1.74×10^−8^). Observation of litters showed partial loss of progeny with death in the first 48–72 hours and some complete loss of litters either abandoned or cannibalized by Ts65Dn mothers. Heart and great vessels microdissection and cardiac histology analyses showed one out of 18 Ts65Dn dead pups presenting both great vessel and cardiac malformations (Figure 1). In this Ts65Dn pup, the right subclavian artery arose from the distal part of the aortic arch downstream the left subclavian artery connection and described a retro-oesophagian loop instead of arising from the proximal part of the aortic arch forming a common trunk with the right carotid artery. Histology of this pup also showed an inter-ventricular communication (Figure 1D) while ventricular septation was complete in all wt pups (Figure 1C). In adults, histological analysis of the heart (hematoxylin-eosin staining) showed no particular differences in trisomics and controls. Fibrosis was checked in 3 individuals of each group with trichrome coloration but no specific alteration and no large fibrosis patch could be noticed. We conclude that in our breeding colony, the Ts65Dn early post-natal death is associated with a low-incidence of cardiac malformations similar to those of previous reports [14], [15].

**Table 1:** Transmission rates of the Ts65Dn and Ms5Yah alleles observed at weaning in Ts65Dn, Ms5Yah, and Ts65Dn/Ms5Yah mice.

*Original cross*	*Genotype*	*Observed number*	*Observed ratio*	*χ^2^*	*P*
Ts65Dn x F1B6C3B	wt	407	66.0%	31.8	1.74×10^−08^
	Ts65Dn	210	34.0%		
Ms5Yah x F1B6C3B	wt	85	83.3%	23.5	1.27×10^−06^
	Ms5Yah	17	16.7%		
Ts65Dn x Ms5Yah	wt	73	39.5%	8.51	3.55×10^−3^
	Ts65Dn	50	27.0%	0.01	0.90
	Ms5Yah	16	8.6%	0.27	0.60
	Ts65Dn/Ms5Yah	46	24.9%	24.0	9.77×10^−07^

All the lines were kept on a mixed B6C3B background. Ts65Dn and Ms5Yah lines showed a reduced transmission rate of their respective allele at weaning that was related to post-natal lethality. Combination of both aneuploidies leads to a partial rescue for this lethality as shown by the ratio observed for the Ts65Dn/Ms5Yah genotype.

### Abnormal electrocardiologic pattern in Ts65Dn

**Figure 2:**
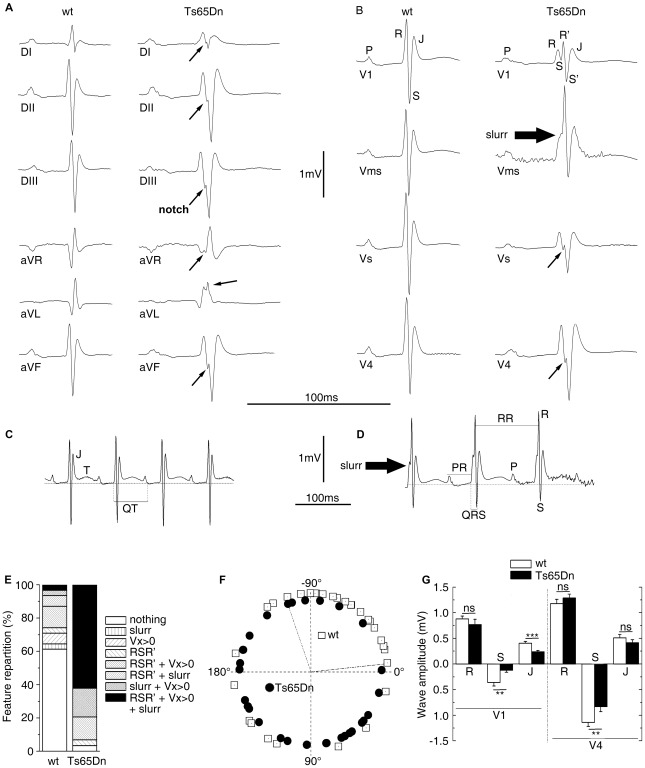
ECG analyses of urethane anaesthetized Ts65Dn adult mice revealed cardiac conduction anomalies. Peripheral (A) and precordial (B) leads are presented for the wt and the Ts65Dn mice in the left and right columns respectively. Wt ECG showed a smooth tracing in all leads. P wave, QRS complex and J wave are clearly distinguishable. Ts65Dn ECG showed notching of the S wave in most leads. In V1, QRS amplitude was reduced with a fragmented RSR'S' complex. A slurr was found in the Vms lead. Notice the prolonged PR interval. Representation of Vms lead from wt (C) and Ts65Dn (D) showed measurements performed on wave amplitudes and durations. The isoelectric line (zero) was taken as the mean potential just preceding the R wave and J and T waves were labeled. The T wave was always positive in this lead while it could be elusive in other leads. ECG features (E) were classified according to the presence of fragmented QRS (RSR'S'), slurr and S wave absence (Vx>0) in the precordial leads. 62% of the Ts65Dn mice showed 3 anomalies while 61% of the wt was free of such features. Frontal QRS electrical axis distribution (F) was evenly distributed in Ts65Dn while that of wt animals showed a leftward preferential orientation. Measurement of wave amplitudes in precordial leads (G) showed a large decrease of S and J waves on V1 and limited changes in V4 whereas R waves were found normal in both V1 and V4 (n = 31 and 29 for wt and Ts65Dn respectively) (Student t-test p-values: **: *P*<0.01; ***: *P*<0.001).

Since DS patients can show electrocardiographic and functional changes even in the absence of overt CHD, an electrocardiographic investigation of Ts65Dn mice was performed to look at more subtle phenotypes than gross morphological anomalies. The six standard peripheral leads (DI, DII, DIII and aVR, aVL, aVF) ECG of wt and Ts65Dn mice are illustrated in Figure 2. In addition we defined four new precordial leads (V1, Vms, Vs and V4) with respect to the Wilson reference terminal (Figure 2). Recording from each lead showed a typical mouse ECG including P and R waves and characterized by a large S wave followed by a J wave. A delayed T wave, either positive or negative, was often elusive except for the Vms lead in which it was always present and positive as illustrated in Figure 2C and 2D. However, while P, QRS and JT waves showed a smooth time-course in wt mice, QRS exhibited a fragmented time-course in Ts65Dn mice (Figure 2A and 2B). In most leads, QRS wave showed a notch while V1 precordial lead exhibited an obvious RSR' or RSR'S' shape with a noticeable decrease of the S wave amplitude. Vms showed a slurring at the foot of the R wave. Fragmented QRS (fQRS) with notch were most frequent in the inferior frontal leads (DII, DIII, aVF) and RSR' with low voltage S wave and slurring were more frequent in V1 and Vms precordial leads while V4 was less affected. Relative distribution of these features in wt and Ts65Dn are presented in Figure 2E showing that 63% of Ts65Dn mice are affected by three of these anomalies at a time compared to less than 3% in wild-type (wt). Fisher exact test was performed on these qualitative features of the ECG as reported in Table 2 taking into account the presence of at least two of these features. Ts65Dn ECG are highly significantly different from those of wt mice (Fisher exact test, P = 2×10^−5^). Moreover, these ECG features were found to be highly predictive of a Ts65Dn genotype.

**Table 2:** Comparison of ECG anomalies in the different mouse lines.

				Fisher exact test (2 tailed)	
Genotype	2 features	1 or 0 feature	Total	versus wt	versus Ts65Dn
wt	8 (25%)	23 (75%)	31		
Ts65Dn	27 (93%)	2 (7%)	29	P = 2.2×10^−5^	
Ts65Dn/Ms5Yah	4 (33%)	8 (67%)	12	P = 0.444	P = 1.8×10^−4^

ECG anomalies were featured by RSR'S' fragmented QRS waves. low voltage S wave in V1 and/or Vms. and presence of a slurr in V1 or Vms. Values correspond to the number of mice with the proportion given in parentheses.

Noticeable changes in the wave amplitudes were also recorded in Ts65Dn as compared with wt (Table 3). In the frontal plane, inferior lead QRS amplitude decreased by 25% for the R wave and by up to 40% for the S wave while QRS amplitude on superior leads increased by 7% for the R wave (non statistically significant) and 80% for the S wave. Overall changes in waves amplitudes resulted in large changes of the frontal ventricular electrical axis as illustrated in Figure 2F. While 70% of the wt mice axis is within −7° to −124°, most of the Ts65Dn axis (79%) are outside this range (Fischer exact test, P = 1.1×10^−4^). Figure 2G shows in addition that Ts65Dn ventricular electrical axis is evenly distributed in contrast to wt. In the frontal plane, P wave amplitudes changes followed that of the QRS. They showed a slight decrease in the inferior lead while there was no significant change in the superior leads.

**Table 3:** P, R, and S wave amplitudes (mV) of wt and Ts65Dn mice (n = 18) for superior leads (DI and aVR), inferior leads (DII, DIII, and aVF), and precordial leads (V1, Vms, Vs, and V4).

Waves	P		R		S	
lead	wt	Ts65Dn	wt	Ts65Dn	wt	Ts65Dn
DI	0.081±0.005	0.085±0.008	0.334±0.034	0.356±0.037	−0.187±0.028	−0.347±0.033**
-aVR	0.138±0.007	0.120±0.017	0.446±0.031	0.332±0.047*	−0.331±0.024	−0.401±0.026*
DII	0.172±0.009	0.159±0.009	0.670±0.036	0.545±0.039*	−0.662±0.039	−0.527±0.044*
DIII	0.093±0.008	0.090±0.018	0.555±0.031	0.433±0.029**	−0.793±0.067	−0.504±0.071**
aVF	0.128±0.008	0.107±0.010*	0.622±0.033	0.468±0.032**	−0.756±0.051	−0.452±0.053**
V1	0.107±0.009	0.073±0.018*	0.879±0.056	0.770±0.104	−0.358±0.070	−0.119±0.041**
Vms	0.181±0.010	0.151±0.013*	1.574±0.106	1.466±0.157	−0.723±0.096	−0.291±0.156**
Vs	0.117±0.009	0.097±0.011*	0.897±0.049	0.716±0.043*	−0.714±0.059	−0.313±0.048***
V4	0.096±0.053	0.161±0.019*	1.178±0.084	1.290±0.075	−1.137±0.079	−0.832±0.097**

Significance is given with P values of 0.05 (*). 0.01 (**) and 0.001 (***).

**Figure 3:**
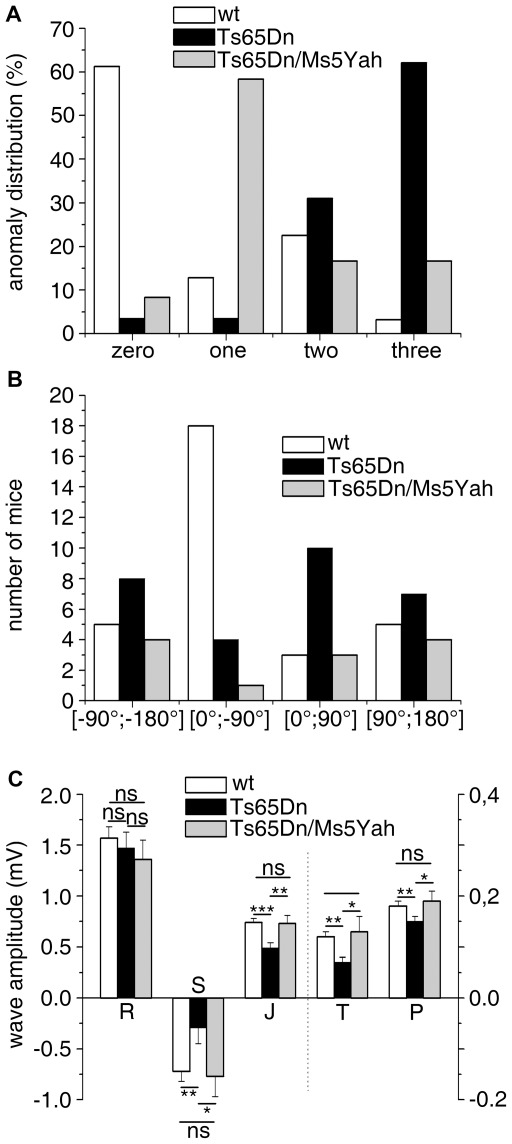
Partial rescue of ECG features in Ts65Dn/Ms5Yah compound mice. (A) ECG features from urethane anaesthetized adult mice were classified according to the presence of fragmented QRS (RSR'S'), slurr and S wave absence (Vx>0) in the precordial leads. 62% of the Ts65Dn mice showed 3 anomalies while 61% of the wt was free of such features. Only 16% of Ts65Dn/Ms5Yah mice exhibited 3 anomalies and 65% had none or only one anomaly. (B) In the frontal plane, the electrical axis of both Ts65Dn and Ts65Dn/Ms5Yah animals were evenly distributed while that of wt showed a leftward preferential orientation. (C) wt, Ts65Dn and Ts65Dn/Ms5Yah ECG wave amplitudes were recorded in the precordial Vms lead. P, S, J and T waves' amplitudes were all reduced in Ts65Dn mice as compared to wt while R wave was not changed. Double transgenic mice (Ts65Dn/Ms5Yah) rescued these effects (Mean ± sem with ANOVA p values ***: *P*<0.001; **: *P*<0.01; *: *P*<0.05 for 29, 18 and 12 wt, Ts65Dn and Ts65Dn/Ms5Yah respectively).

The results were at variance in the precordial leads (Table 3; Figure 2G). R wave amplitude changes were small and non-significant but S wave was significantly reduced by 60 to 70% in V1, Vms and Vs. Figure 2G shows much smaller waves changes in left hand side lead V4 (only 25%) than in right hand side lead V1. P wave was reduced by 20% to 30% in V1, Vms and Vs but increased in V4 by 68% (Table 3). Waves related to the repolarisation process (J and T) were also affected in Ts65Dn compared to wt mice. J amplitude was reduced by 20 to 40% in the inferior leads and in V1, Vms and Vs (Figure 2G). In contrast the change was much smaller in superior leads (aVR, DI, 10%) and V4 (Figure 2G). As illustrated in Figure 3, T wave amplitude was also reduced in Vms. Not only Ts65Dn mice ECG showed changes in wave shapes and amplitudes but they also showed changes in wave durations and intervals. The RR intervals were prolonged in Ts65Dn leading to a reduction of heart rate from 667±11 bpm to 606±13 bpm (n = 30; Student t-test; *P* = 9×10^−4^). PR was prolonged from 35.3±0.5 ms to 44.6±1.1 ms (Student t-test; *P* = 7×10^−5^). QRS wave duration was also increased by 8%. Values were respectively 9.02±0.21 ms in wt and 9.89±0.2 ms in Ts65Dn (Student-t-test; *P* = 0.027). QT and QTc were also larger in Ts65Dn (73.0±2.0 ms and 73.0±1.3 ms respectively) than in wt (67.5±1.0 ms and 71.0±0.1 ms; Student t-test; *P* = 0.004 and 0.02). Since the above-described results point to conduction changes in Ts65Dn as compared to wt, a sub-group of these mice (N = 8) were treated with the Na channel blocker, flecainide. As reported in Table 4, flecainide (20 mg/kg) significantly prolonged all intervals and wave durations (P, R and QRS waves and RR, PR and QT intervals). With this treatment, adult Ts65Dn showed a specific electrophysiologic signature as compare to wt mice. Not only was the wave-front activation changed but a first degree conduction bloc and alteration of the repolarisation were also recorded in the DS mouse model. Thus the investigation of Ts65Dn mice revealed several electrocardiographic phenotypes even in the absence of gross morphological anomalies in this DS model.

**Table 4:** Effect of the sodium channel blocker flecainide on the duration (ms) of P, R, and QRS waves and RR, PR, QT, and QTc intervals in Ts65Dn mice (N = 8).

Interval/wave	control	Flecainide
RR	94.00±2.69	111.88±2.92***
PR	38.50±1.67	55.63±2.23***
P	11.75±0.53	21.13±2.31***
R	6.50±0.68	14.25±0.77***
QRS	9.63±0.32	14.25±0.77***
QT	66.57±1.13	81.88±2.58**
QTc	68.69±1.38	77.49±2.33**

Significance is given between control and flecainide with *P* values of 0.05 (*). 0.01 (**) and 0.001 (***).

### Early post-natal lethality of Ts65Dn mice is partially rescued by the Ms5Yah monosomy of the *App-Runx1* interval

The Ts65Dn allele induces early post-natal lethality which has also been described in Ts1Yu mice, a trisomic model for a larger region of Mmu16 extending from *Lipi* to *Zfp295* [16]. Such a lethality was not found in the Ts1Cje mouse model which is trisomic for a smaller segment from *Sod1* to *Zfp295*, nor in the Ts1Rhr carrying a trisomy from *Cbr1* to *Orf9* [3], [4], [20]. Accordingly we tested if the early post natal lethality was associated with 3 copies of the *Mrpl39-Sod1* interval. Thus we developed a partial monosomic model named Ms5Yah carrying a 7.7 Mb deletion on Mmu16 between the *App* and *Runx1* genes (Figure S1). The transmission rates at weaning for Ms5Yah allele and the combination between Ts65Dn and Ms5Yah (Ts65Dn/Ms5Yah) alleles are reported in Table 1. From the 102 individuals alive at weaning in the Ms5Yah breeding colony, 16.7% of the mice carried the *App*–*Runx1* deletion. This transmission rate for the partial monosomy is below the Mendelian ratio (χ^2^ *P* = 1.26×10^−06^) indicating that the region causes lethality due to haploinsufficiency. Interestingly the transmission rate was evaluated at 27.9% at post natal day 1 (N = 43, χ^2^ *P* = 1.2×10^−03^) and 48.3% at embryonic day 18.5 (N = 31, χ^2^ *P* = 0.85) indicating a lethality within this period. Thus we considered the *App-Runx1* region as a haploinsufficient region inducing post-natal lethality. We checked whether the Ms5Yah pups were carrying cardiovascular defects as observed in the Ts65Dn newborns using microdissection and histological analyses. No cardiovascular malformations, either in the great vessels or in the intra-cardiac septation could be seen in 18 dead pups. 185 Ts65Dn/Ms5Yah offspring were obtained at weaning from the breeding of Ms5Yah males with Ts65Dn females. The expected ratios were calculated considering the previously described transmission rates obtained for Ts65Dn line and Ms5Yah line. Transmission ratios for Ts65Dn and Ms5Yah alone were not significantly different from the expected ratio (27.0% and 8.6% respectively, χ^2^ *P*>0.05). However, the transmission rate for combined Ts65Dn/Ms5Yah alleles was 24.9%, as expected for a Mendelian ratio showing that the Ts65Dn-allele-induced lethality was completely rescued by the monosomy of the *App-Runx1* region (χ^2^ *P* = 4.8×10^−7^). However the transmission rate for the Ms5Yah allele did not reach the expected value showing that Ms5Yah-induced lethality still occurs. Thus the deletion between *App* and *Runx1* genes (Ms5Yah) induced a severe postnatal lethality that is partially rescued by combining Ms5Yah with the Ts65Dn model suggesting effect due to the new chromosomal configuration.

### Combination between Ms5Yah and Ts65Dn models rescues part of Ts65Dn ECG phenotypes

As we found that the Ms5Yah monosomy rescued Ts65Dn post-natal lethality, we decided to look at ECG in Ts65Dn/Ms5Yah adult mice to determine whether the combination between these two models had an effect on electrocardiologic pattern. Ts65Dn/Ms5Yah mice showed wave shape anomalies similar to those described above for Ts65Dn mice. However, relative distribution of these features in Ts65Dn/Ms5Yah (Figure 3A) showed a noticeably lower rate of notching in the lower frontal leads as well as a reduced frequency of RSR'S' waves in V1 and Vms as compared with Ts65Dn. Taking into account the presence of at least two wave shape features, Ts65Dn/Ms5Yah mice could no longer be distinguished from wt and were significantly different from the Ts65Dn mice (Table 2) supporting an overall rescue-like effect of the Ms5Yah allele. This occurred because Ts65Dn features no longer cumulated and were reduced to a single anomaly in 65% of the Ts65Dn/Ms5Yah individuals

As Ts65Dn mice showed a variable orientation of the QRS axis, this item was also determined for Ts65Dn/MS5Yah mice. Repartition of this axis for wt, Ts65Dn and Ts65Dn/Ms5Yah by quadrants is reported in Figure 3B. Most wt mice showed an electrical axis in the 0°;−90° quadrant (upper right) while Ts65Dn ventricular electrical axis was evenly distributed. No obvious rescue of the electrical axis was found in Ts65Dn/Ms5Yah mice. 75% of the axis of the latter mice stayed outside the −7°;−124° interval previously mentioned with a median value of +162°. This significantly differed from the wt (Fisher exact test, *P* = 0.014) but not from the Ts65Dn (*P* = 0.53).

In the frontal plane, wave amplitude of Ts65Dn/Ms5Yah mice showed the same trend as Ts65Dn mice when compared with wt, with significant decrease of the S wave amplitude in inferior leads, increase in superior leads and smaller changes in R waves. While the Ts65Dn/Ms5Yah model did not rescue the changes in wave amplitudes in the frontal plane, it completely rescued changes in the sagittal plane (precordial leads) as illustrated in Figure 3C for the Vms lead. With the exception of the S wave in the V1 lead, none of the precordial P, R, S, J and T waves of Ts65Dn/Ms5Yah mice was different from those of wt mice in each of the four precordial leads. The rescue holds not only for the ventricular S wave but also for the auricular P wave and for the J and T waves related to repolarisation in the precordial leads. In these mice RR (97.8±4.0 ms) and PR (39.3±4.5 ms) were shorter than in Ts65Dn mice but these values did not differ significantly from either wt (*P* = 0.23 and *P* = 0.052) or Ts65Dn (*P* = 0.57 and *P* = 0.48) mice. QTc was significantly reduced down to 65.5±2.8 ms (*P* = 0.03; n = 12) in Ts65Dn/Ms5Yah mice. This value was no longer different from the wt value (*P* = 0.075). Thus two copies of the *App-Runx1* region in Ts65Dn/Ms5Yah mice led to a partial recovery of the Ts65Dn electrocardiographic phenotypes. Wave shape anomalies, wave amplitudes on precordial leads and QTc duration were rescued whereas wave amplitudes on peripheral leads were not and RR and PR were partially rescued as they showed an intermediate duration between Ts65Dn and wt values.

### Changes in whole-genome expression in Ts65Dn and compound mutant hearts

**Figure 4:**
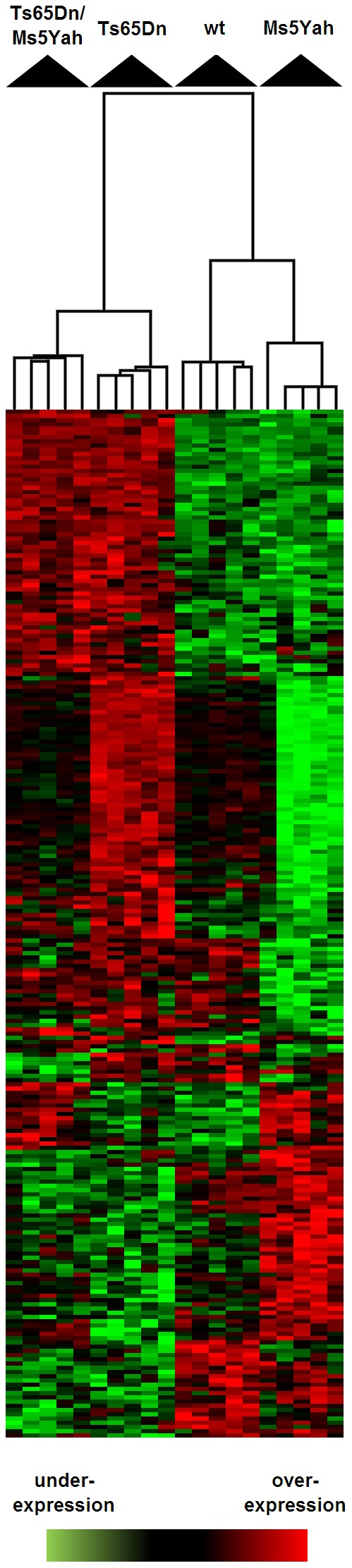
Clustering derived from statistically deregulated genes in Ts65Dn, Ms5Yah, and Ts65Dn/Ms5Yah. Whole genome microarray expression analysis was performed on RNA isolated from whole adult mouse hearts and statistically deregulated genes were assessed using GeneSpring software. Clustering showed proximity between Ts65Dn/Ms5Yah and Ts65Dn arrays on one side, and between wt and Ms5Yah arrays on the other side. Under-expression and over-expression are represented in green and red respectively, expression levels were calculated by comparison to mean expression level of all arrays for each gene.

The rescue of Ts65Dn viability and electrocardiographic phenotypes by reestablishing the *App-Runx1* region to two copies in Ts65Dn mice points at the gene dosage effect related to this interval. Whole genome expression arrays were then performed on adult mice heart samples to determine genes that are dosage sensitive and to observe the deregulations on the whole genome. Wt, Ts65Dn, Ms5Yah and Ts65Dn/Ms5Yah heart samples were analysed using Affymetrix Gene Chip technology. RMA normalized data obtained from Affymetrix Expression Console software were filtered using an expression level threshold above 50 in raw data (5.644 in log scale 2) and fold change (FC) between wt and transgenic mice >1.2 or <0.8. We measured the expression levels of 25,099 transcripts, representing 22,193 genes. Among those, 9,017 genes (40.6%) had a fluorescence signal above the threshold in wt samples and were considered as expressed genes in adult mouse heart. The ratio of gene expression across chromosomes between aneuploid and wt mice was 1.00±0.002 (ranging from 0.98±0.003 to 1.03±0.005) except for genes located on the monosomic and trisomic intervals located on Mmu16 and Mmu17 for the specific region found in the Ts65Dn minichromosome [20] (Figure 4A; see below).

In order to further analyze gene expression modifications GeneSpring software was used to define the most significantly deregulated genes using one way ANOVA and a Tukey HSD *post hoc* analysis. The clustering on arrays of most significantly deregulated genes (ANOVA, *P*<0.1) are represented on Figure 4. A partial correlation was observed between Ts65Dn/Ms5Yah and Ts65Dn arrays on one hand and between wt and Ms5Yah arrays on the other hand as shown by the upper tree. This is due to the number of genes in common between Ts65Dn and Ts65Dn/Ms5Yah on the one hand and between wt and Ms5Yah on the other hand. The deregulation of 5 genes of the Mmu16 was confirmed by QRT-PCR in the heart with different genotypes and confirmed the results of the microarray analysis (Table 5).

**Table 5:** Ratio of the expression level of 5 genes located on Mmu16 in the heart of mutant compared to disomic control mice.

Gene	Ts65Dn	Ts65Dn/Ms5Yah	Ms5Yah
*Usp16*	1.68±0.21*	1.16±0.30	0.54±0.16*
*Cct8*	1.72±0.14**	1.01±0.14	0.44±0.09**
*Bach1*	1.77±0.31**	1.10±0.22	0.58±0.16*
*Dyrk1a*	1.57±0.12**	1.64±0.20**	1.06±0.10
*Sh3bgr*	1.68±0.22*	1.57±0.27*	0.74±0.11

The analysis was done by qRT-PCR. Significance is given with *P* values of 0.05 (*). 0.01 (**).

Gene expression profiles were categorized in seven distinct groups with respect to genotype-associated expression patterns that are listed in Table 6 and Table S1. The first group was mainly composed by triplicated genes in both Ts65Dn and Ts65Dn/Ms5Yah mice, either located on Mmu16, upstream of *App* or downstream of *Runx1*, or on the Mmu17 centromeric regions of the Ts65Dn minichromosome. These genes were found over-expressed in both Ts65Dn and Ts65Dn/Ms5Yah arrays but not in Ms5Yah arrays. We also found a set of 12 genes located elsewhere whose expression follow the same rationale, 9 overexpressed and 3 down-regulated as shown on Table 6. These genes might correspond to targets of the triplicated genes in the Ts65Dn/Ms5Yah mice. A second group of genes was mainly composed by genes located within the Ms5Yah region and were found over-expressed in Ts65Dn, under-expressed in Ms5Yah and with comparable expression level in wt and Ts65Dn/Ms5Yah arrays. Interestingly we found one gene from the telomeric part of Mmu16, *Msx2*, and only 6 additional genes from different chromosomes that had similar expression variations correlated with changes both in the number of copy of the *App-Runx1* region and in the Ts65Dn trisomy. The other groups were mainly composed of genes located outside the above-mentioned Mmu16–Mmu17 regions and were distinguished by their expression patterns. Genes from the third group were differentially expressed in Ts65Dn arrays, while comparable to wt in Ms5Yah and in Ts65Dn/Ms5Yah arrays. 3 genes from this group were found in the proximal region of Mmu17 that is trisomic in the Ts65Dn model and one, *Tiam1*, was located in the *App-Runx1* region but whose expression is not sensitive to dosage in Ms5Yah mice. The expression of genes from the fourth group was modified only in the Ms5Yah model. Among those genes, one gene, *Sft2d1*, comes from the centromeric region of Mmu17 and 5 genes (*Ifngr2, 1110004E09Rik, 2610039C10Rik, Sod1* and *Atp5o*) are from the Ms5Yah interval. They were all down-regulated. In addition 13 down-regulated genes and 18 up-regulated genes are found outside the aneuploid regions. A fifth group contained 10 genes over-expressed in Ms5Yah and Ts65Dn/Ms5Yah arrays whereas 15 additional genes displayed a more complex expression pattern (groups 6 and 7).

**Table 6:** Genes deregulated in the heart of Ts65Dn, Ms5Yah, and Ts65Dn/Ms5Yah compound mice.

		Genotype		Ts65Dn	Ms5Yah	Ts65Dn/Ms5Yah
Group	Gene Name	Chr	Anova	mean ± sem	mean ± sem	mean ± sem
1	*AU021092*	16	**	1.26±0.11*	0.87±0.04	1.32±0.1**
	*Heph*	X	***	1.27±0.06*	1.11±0.05	1.43±0.1**
	*Fbln2*	6	**	1.3±0.08**	0.96±0.03	1.34±0.08***
	*AI593442*	9	**	1.83±0.39**	0.94±0.06	1.68±0.25**
	*Pdgfd*	9	*	1.23±0.02**	0.94±0.04	1.21±0.08*
	*F5*	1		1.49±0.14**	1.08±0.12	1.22±0.05
	*Vwf*	6		1.32±0.08**	1.02±0.08	1.27±0.08**
	*Pcbd1*	10		1.66±0.22**	0.86±0.1	1.34±0.3
	*P2ry12*	3		1.26±0.08**	1.01±0.06	1.22±0.07**
	*Lrp2bp*	8		0.65±0.04***	0.83±0.13	0.64±0.07***
	*Htr2a*	14		0.7±0.06***	0.83±0.07	0.67±0.1**
	*Clk1*	7		0.77±0.05*	0.85±0.05	0.72±0.03**
2	*Irf7*	7		1.37±0.26*	0.67±0.13	0.93±0.13
	*Zfp583*	7		1.29±0.1**	0.72±0.15	0.99±0.05
	*Slc38a4*	15	**	0.76±0.06***	1.27±0.14	0.86±0.07
	*Slc8a1*	17		0.8±0.07	1.34±0.14	1.03±0.11
	*Adam19*	11	*	1.28±0.2	1.89±0.34*	1.06±0.09
	*Vmn2r86*	10	*	1.59±0.22**	1.38±0.13**	1.1±0.12
3	*Wrb*	16	***	1.22±0.02***	0.89±0.02	1.18±0.04
	*St8sia5*	18		1.27±0.14**	1.19±0.08	1.18±0.06
	*Slc39a6*	18		1.25±0.11**	0.97±0.04	1.14±0.08
	*Abi3bp*	16		1.42±0.11**	1.03±0.1	1.2±0.06
	*BC055004*	5		1.5±0.22**	0.84±0.07	1.14±0.23
	*Dyrk1b*	7	*	0.73±0.04***	0.82±0.02	0.84±0.03
	*Ypel2*	11	*	0.79±0.06	1.18±0.1	0.9±0.1
	*Chrna2*	14		0.78±0.1**	0.8±0.04	0.81±0.09
	*Ldhd*	8		0.79±0.05**	0.93±0.03	0.86±0.03
	*Gm16493*	9		0.71±0.07*	1.01±0.07	0.89±0.06
	*Pm20d1*	1		0.65±0.08***	0.96±0.1	0.88±0.12
	*Nudt8*	19	*	0.79±0.05**	1.05±0.04	0.94±0.01
4	*Carkd*	8	*	0.93±0.04	0.79±0.01**	0.91±0.05
	*Amot*	X	*	1.02±0.12	0.72±0.06*	1.01±0.02
	*Pnck*	X	*	0.94±0.09	0.64±0.09**	0.85±0.02
	*Gm8566*	6	*	1.13±0.05	0.74±0.1**	0.96±0.01
	*Gm5436*	12	**	1.14±0.1	0.71±0.09*	0.97±0.03
	*Hmcn2*	2	*	1.12±0.13	0.78±0.06	1.19±0.11
	*Scn4b*	9	**	1.06±0.22	0.34±0.08*	1.12±0.18
	*Rasl10b*	11	*	0.9±0.05	0.72±0.06	1.02±0.07
	*Tnxb*	17		0.96±0.1	0.75±0.05	0.96±0.05
	*Pde7b*	10		0.87±0.07	0.73±0.03*	0.9±0.08
	*C1qa*	4		1.01±0.08	0.74±0.07	1.07±0.06
	*Ccne2*	4		0.99±0.07	0.71±0.06*	0.82±0.07
	*Crnkl1*	2		0.92±0.04	0.79±0.01	0.85±0.04
	*Cerk*	15	*	1.11±0.1	1.29±0.06	0.94±0.03
	*2810410L24Rik*	11		0.94±0.07	1.24±0.11	1.14±0.1
	*Gm10400*	6		0.86±0.09	1.28±0.09	0.91±0.1
	*2210021J22Rik*	15		1.05±0.07	1.35±0.02	1.08±0.08
	*Fam189a1*	7		1.01±0.07	1.23±0.08	1.15±0.05
	*Adamts6*	13		1±0.06	1.36±0.18	1.01±0.08
	*Zc3h12c*	9		1.09±0.06	1.3±0.03	1.08±0.06
	*Lrrfip1*	1		1.02±0.06	1.25±0.03	1.05±0.05
	*Lce1m*	3		1.07±0.05	1.25±0.06	1.15±0.09
	*Fzd3*	14		1.1±0.07	1.21±0.09	0.93±0.06
	*Pnrc1*	4		1.02±0.04	1.33±0.05	1.07±0.05
	*Tnni1*	1	*	1.01±0.1	1.34±0.1**	1.13±0.05
	*mIR697*	4	*	0.99±0.03	1.25±0.09**	1.06±0.06
	*St3gal4*	9	**	1±0.05	1.27±0.03***	1.15±0.08
	*Mapk4*	18	*	0.86±0.07	1.28±0.13*	0.91±0.07
	*Ybx2*	11	*	0.82±0.09	1.23±0.13*	0.96±0.02
	*Cass4*	2	*	1.02±0.05	1.25±0.09**	1.16±0.03
	*Clec12b*	6	*	0.93±0.06	1.33±0.1*	1.05±0.08
5	*Ccl24*	5	***	0.99±0.14	2.13±0.38**	1.31±0.11
	*ENSMUSG00000073686*	4	*	1±0.04	1.22±0.06***	1.23±0.08**
	*Olfr234*	15	*	1.09±0.07	1.39±0.16**	1.38±0.07***
	*Disp2*	2	*	0.95±0.07	1.33±0.12**	1.26±0.07**
	*Mmp9*	2		0.99±0.11	1.78±0.25	1.53±0.34
	*Tnrc4*	3		1.04±0.05	1.29±0.06	1.28±0.12*
	*Loxhd1*	18		1.14±0.11	1.32±0.09	1.2±0.06**
	*Adamts18*	8		1.1±0.07	1.23±0.06	1.34±0.12**
	*mIR125a*	17		1.13±0.05	1.27±0.06	1.22±0.08**
	*Grin2b*	6	**	1.15±0.03	1.23±0.04***	1.26±0.03***
6	*Plvap*	8	*	1.59±0.17**	1.34±0.03***	1.42±0.1**
	*S100a11*	3		1.43±0.11**	1.21±0.1	1.4±0.07**
	*Kctd15*	7		1.21±0.05**	1.3±0.08	1.3±0.09**
	*Abcd3*	3		0.77±0.06**	0.79±0.06*	0.75±0.05***
	*Tubb1*	2		1.93±0.2**	1.17±0.15	1.5±0.13
	*Ppbp*	5	*	5.27±1.51**	1.75±0.38	3.45±0.75**
7	*Irs3*	5	**	0.94±0.05	1.09±0.07	1.21±0.05***
	*Gon4l*	3		0.84±0.06	0.91±0.05	0.79±0.05***
	*Sesn3*	9		0.85±0.06	0.87±0.04	0.79±0.05***
	*Fam163a*	1		1.07±0.05	1±0.06	1.21±0.07**
	*Slco3a1*	7		1.03±0.04	1.14±0.03	1.2±0.09**
	*Aard*	15		1.07±0.04	1.12±0.04	1.2±0.05***

Only the genes for which the Anova test is below 0.1 and which are not triplicated on the Ts65Dn minichromosome are listed here. (*P* values: ***: *P*<0.001; **: *P*<0.01; *: *P*<0.05).

**Figure 5:**
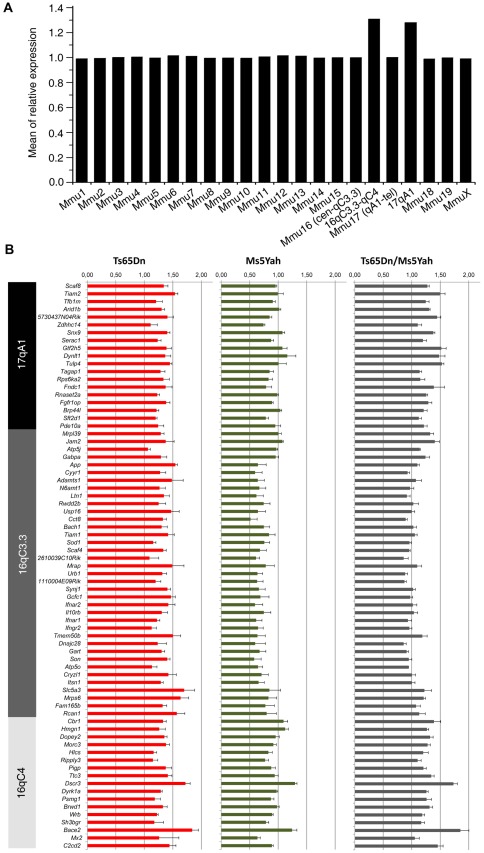
Expression profile of genes located on the Ts65Dn minichromosome obtained from whole-genome microarray expression analysis from whole adult mouse hearts. (A) Mean gene expression ratios between Ts65Dn and wt mice according to chromosomal location showed an increase for genes located on the Ts65Dn minichromosome (Mmu16 and 17). (B) Differences in the expression of genes present on the Ts65Dn minichromosome in the different mouse models and showing ∼1.5 fold over expression in the Ts65Dn model, ∼0.5 fold downregulation of genes between *App* and *Runx1* (located upstream of *Cbr1*) in Ms5Yah mice with a normal expression of other Ts65Dn minichromosome genes, and up-regulation comparable to Ts65Dn except for genes between *App* and *Runx1* that are found expressed normally in Ts65Dn/Ms5Yah mice. Hence combination of Ms5Yah and Ts65Dn constructions led to gene expression rescue within the *App*–*Runx1* region.

Looking more closely at the 109 Mmu16 known protein coding genes present on the Ts65Dn minichromosome, we found that 4 were absent from the chip and 39 were expressed below background level. Among the 66 remaining genes expressed in adult mice heart, 78.8% were up-regulated in Ts65Dn arrays with a mean fold change of 1.36±0.01 (ranging from 1.20 to 1.61). Similarly, of the 43 Mmu17 known protein coding genes located on the Ts65Dn minichromosome, 8 were absent from the chip and 7 were not expressed. Of the 28 Mmu17 remaining trisomic genes expressed in adult mice heart, 78.6% were upregulated in Ts65Dn arrays with a mean fold change versus wt of 1.36±0.02 (ranging from 1.21 to 1.55). Thus the presence of the Ts65Dn minichromosome results in an overall upregulation of genes present in three copies as shown on Figure 5. Interestingly Ts65Dn/Ms5Yah arrays showed an overall upregulation profile close to that of the Ts65Dn arrays, except for genes located within the *App*-*Runx1* region that were expressed at a ratio versus wt close to 1.0. As shown in Figure 5, some genes do not seem to be dosage sensitive and show no change versus wild type in all aneuploid samples. Actually most of these genes (22 located on Mmu16 and 8 on Mmu17) showed a raw expression below background level and are in fact not expressed in adult mice hearts. Nevertheless five genes located on Mmu16 (*Atp5j*, *Cldn14*, *Erg*, *Pcp4* and *Prdm15*) and four on Mmu17 (*Pisd-ps2*, *Zdhhc14*, *Synj2* and *Prr18*) were found expressed but not dosage sensitive in heart.

Five genes were selected to validate expression levels observed with arrays using quantitative PCR analysis (Figure 5B, Table 6). *Usp16*, *Cct8* and *Bach1* (located between *App* and *Runx1* genes) showed fold change versus wt above 1.2 in Ts65Dn, below 0.8 in Ms5Yah and close to 1.0 in Ts65Dn/Ms5Yah samples. *Dyrk1a* and *Sh3bgr* (located between *Runx1* and *Zfp295*) showed over-expression (fold change above 1.2) in Ts65Dn and Ts65Dn/Ms5Yah and fold change close to 1.0 (*Dyrk1a*) and slightly under-expressed (*Sh3bgr*) in Ms5Yah samples. All these data confirm the results obtained from expression arrays. Thus variation of gene expression was scored and partly verified for several genes which belong to groups of genes misregulated as a consequence of Ms5Yah or of Ts65Dn aneuploidies. Some of them were located outside the aneuploid regions, indicating a genome-wide trans effect on gene expression in Ts65Dn, Ms5Yah andTs65Dn/Ms5Yah mice hearts.

### Connexins and sodium channels expression is altered in the Ts65Dn mouse

To go further in the understanding of ECG anomalies, an additional experiment was designed by targeting and measuring expression levels of genes related to conduction anomalies in ventricles and atria separately. Action potential propagation in the cardiac cellular network mostly depends on three factors: geometry (i.e. His-Purkinje system, fibrosis & morphology), cardiac connexins and ion channels availability, in particular sodium channels [21]. ECG recordings point to altered conduction in aneuploid mice, and no evident trace of fibrosis could be found (data not shown). Thus we decided to obtain anatomical observations of the His-Purkinje system in Ts65Dn hearts using the Cx40^eGFP^ transgenic mouse model whose heart conduction system is labeled with Green Fluorescent Protein (GFP) [22]. Three Cx40^eGFP^/+;Ts65Dn mice affected by clearcut ECG phenotypes were compared to three Cx40^eGFP^/+ disomic mice showing no ECG anomaly. Left and right ventricles were dissected out as described by Miquerol *et al.* [22]. Right and left His bundles specifically labeled by GFP in the ventricle showed characteristic strong fluorescence and gave rise to a dense network of Purkinje fibers. Gross anatomical observation with stereomicroscope showed no obvious differences between wt and Ts65Dn groups (data not shown). Three additional Ts65Dn and 14 wt hearts with various ECG anomalies were dissected and showed no other visible structural differences than inter-individual variations as described by Miquerol *et al.* [22].

**Figure 6:**
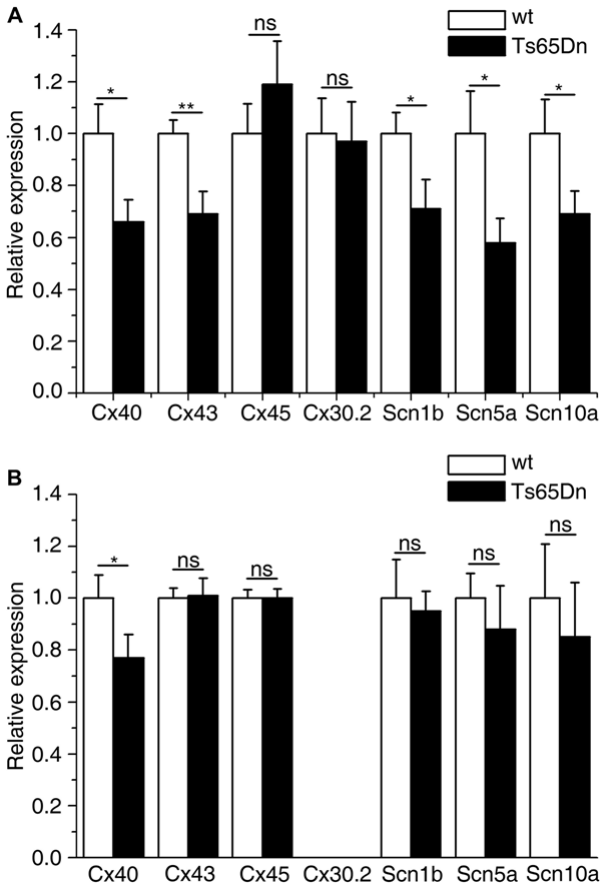
Atrial and ventricular connexins and sodium channel genes expression levels in wt and Ts65Dn mice. (A) Atrial; (B) ventricular. Connexins and sodium channels genes expression levels were assessed by qRT-PCR using RNA extracted from adult mice cardiac atria and ventricle separately. Ts65Dn show an overall decrease in connexin and sodium channel mRNA expression except for *Cx45* and *Cx30.2* in the atria. *Cx40* expression level is reduced in the ventricles. No amplification was obtained for *Cx30.2* in ventricle extracts confirming its atria specific expression pattern. Mean±sem with Student t-test *P*-values, N = 7 and 5 for Ts65Dn and wt respectively.

Even though no anatomical differences were observed in the His-Purkinje system, alterations in connexins or sodium channels expression could still account for electrophysiological anomalies. Connexins *Cx40*, *Cx43*, *Cx45* and *Cx30.2* are chamber- and tissue- specific in the heart [23] and were analyzed accordingly in both the atria and the ventricle. We looked at the *Scn5a* gene which codes for a pore forming protein and is mutated in human heart conduction diseases (Online Mendelian Inheritance in Man 601144, 113900) and for which heterozygous mutant mice suffer from abnormal heartbeats and defects in the impulse conduction system and also at *Scn10a* recently implicated in heart conduction [24]. The beta-subunit of Na^+^ channel *Scn4b* was found underexpressed in Ms5Yah mice using affymetrix array and we also investigated the other beta-subunit *Scn1b* [25]. Connexin mRNA levels were measured by QRT-PCR. Atria showed a down-regulation of *Cx40* and *Cx43* by 34 and 39% respectively and sodium channels-coding genes *Scn5a* and *Scn10a* were also down-regulated by 42% and 31% respectively and *Scn1b* by 29% (Figure 6A). *Cx45* and *Cx30.2* expression levels were not modified in Ts65Dn atria. In Ts65Dn ventricles, *Cx40* was down regulated by 23% but *Cx43*, *Cx45*, *Scn1b*, *Scn5a* and *Scn10a* expression levels were not significantly changed (Figure 4B). Ts65Dn adult mice showed no obvious anatomical anomalies of the cardiac specific conduction system but a decrease of *Cx40* expression in both atria and ventricles and an atria specific decrease of *Cx43*, *Scn1b*, *Scn5a* and *Scn10a* expression. Thus downregulation of these genes might participate to conduction anomalies observed in Ts65Dn adult mice.

## Discussion

Ts65Dn is the most widely used mouse model of DS and displays a large panel of DS features. The present study confirms previous results observed on this model, in terms of birth defects and CHD [14], [15] and points to additional cardiac dysfunctions. CHDs were observed in dead Ts65Dn pups by Moore et al. [14] who reported 3 out of 36 dead pups (8.3%) showing septal defects. Further analysis reported a frequency of 15.3% among 52 dead neonates having some type of cardiovascular abnormality [15]. Our study showed one out of 18 dead pups (5.5%) presenting CHD. This low frequency is certainly due either to the different genetic background of the mice or most probably to the small number of animals analyzed. The frequencies of CHD observed in the Ts65Dn model are however much lower than the 40% observed in human DS. This difference can result from additional contribution of Hsa21 homologous genes not found in the Ts65Dn model, i.e. located outside of the *Mrpl39-Zfp295* interval. A strong candidate is the *Col6a2* gene located in the Mmu10 homologous region and recently found to interact with *Dscam*, trisomic in the Ts65Dn model, to induce cardiac hypertrophy and generate ASD-like septal defects in mouse [26]. Hence, the full range of CHD defects observed in DS results from a combination of genetic interactions between multiple loci along the Hsa21, supporting the “multigenic” theory in which several overexpressed genes interact to establish the DS phenotypes.

A number of functional heart anomalies have been observed in adult DS patients. But this aspect remained unexplored in mouse models. We therefore characterized cardiac function in Ts65Dn animals and we found 93% of Ts65Dn mice carrying constant, robust, and specific ECG signatures strongly different to that of wt littermates. Ts65Dn adult mice exhibited QRS fragmentation (f-QRS), frontal QRS axis dispersion, decrease in right precordial lead amplitude and P wave changes, replicating in part the defects observed in DS people. PR interval prolongation (first degree AV block) observed in Ts65Dn mice includes auricle, AV node and His bundle conduction time. Gross morphology of the His bundle, as deduced from CX40-GFP labeling, does not appear to be changed. The AV node is more likely to account for PR interval prolongation even though enlargement of the auricle, as suggested by P wave amplitude increase in V4 lead and P wave axis change, could also be involved. In DS patients, such AV block and P wave changes have been recorded [6], [17] and related to the AV node displacement in AVSD [11]. Changes in the electrical axis, duration of the QRS and fragmented QRS, observed in Ts65Dn hearts are all characteristics of abnormal ventricular activation that have been described in Hsa21 trisomy [6], [17]–[19] and more generally in CHD [27]–[29]. S wave amplitude, specifically large in mouse and most likely due to specific His-Purkinje bundle and strands/fasciculae distributions [22], contributes largely to the QRS axis changes. In DS and CHD, different QRS axis orientations are related to either primum or secundum ASD, VSD or AVSD [28], [29] and changes in the activation front. An altered organization of the AV node axis [11], [28] and a conduction defect in the trabecular myocardium and papillary muscles sustaining the valves involving the His-Purkinje system [29] could account for such changes. Fragmented QRS (f-QRS) are predictive of arrhythmias [30], [31], a common complication of DS and CHD [5], [11], [12]. f-QRS with a relatively small increase in the QRS duration preferentially reveals a dys-synchrony secondary to heterogeneous intraventricular activation and uncoordinated depolarization of cardiomyocytes in human [30], [31]. This would have escaped observation without the systematic recordings of the precordial leads. These recordings not only point to large changes in shape and amplitude of right side waves in Ts65Dn but also show that the P wave is reduced in Vms and increased in left lead V4.

Most of the observed effects of the Ts65Dn trisomy on ECG are related to conduction known to depend on membrane excitability, intercellular coupling and tissue architecture [21], [32]. This is well illustrated by the presence of fragmented QRS, QRS axis deviation and PR or QRS increase in various transgenic mice that have mutations in or under-express genes coding sodium channels [33], connexins [34], [35] and transcription factors [36], [37]. However, a combination of two of these factors is usually necessary to impair conduction [23]. Since the Na channel blocker flecainide [38] prolongs all wave durations and intervals in Ts65Dn mice, it appears unlikely that decreased availability of Na channel alone could account for the observed changes in conduction. The concomitant reductions in *Cx40*, *Cx43* and Na channels expression that we observed in the auricle of Ts65Dn mice could account for the first degree AV block supported by the prolongation of the PR interval that includes auricle, AV node and His conduction time. This could also account for the P wave amplitude changes. Enlargement of the auricle as suggested by P wave amplitude increase could also participate to PR prolongation. Both the effect of flecainide and the absence of Na channel expression changes in ventricles do not support a decrease in Na channel availability. In the absence of large patch of fibrosis and of clear-cut change in CX40 distribution in Ts65Dn, f-QRS could be related to local defect in CX40 associated to discrete local increase in collagen. This needs further investigation but could account for the observed loss of *Cx40* expression.

**Figure 7:**
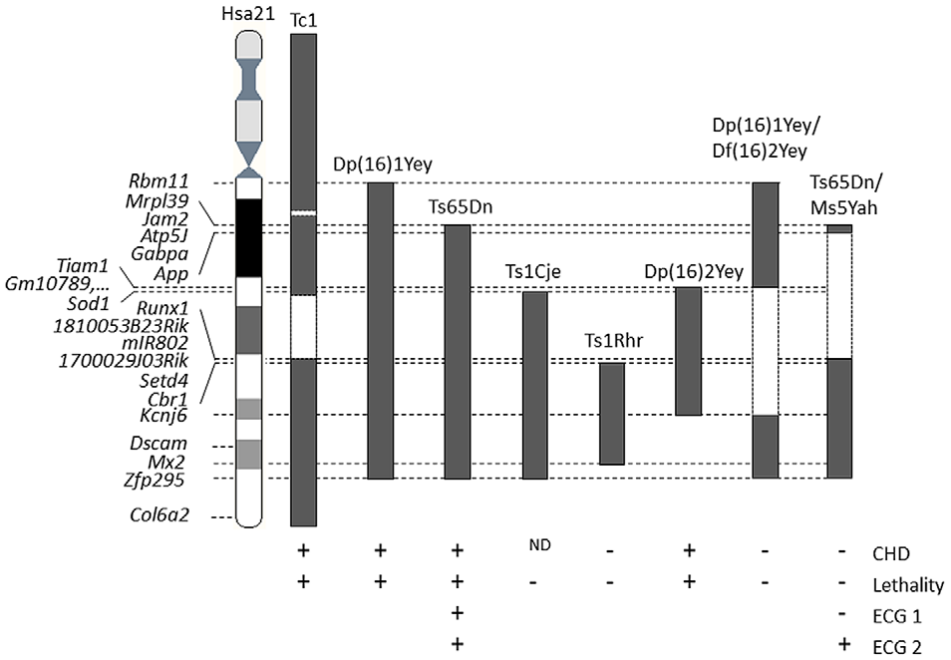
Representation of the DS mouse models displaying lethality and cardiac features. The position of the trisomic segment with the homologous regions to Hsa21, or the Hsa21 regions found in Tc1, is indicated in grey black blocks. While the disomic segments in compound mutants Dp(16)1Yey/Df(16)2Yey or in Ts65Dn/Ms5Yah or the deleted segments in Tc1 are indicated in dashed boxes. “+” indicates the presence and “−” the absence of phenotypes whereas ND indicates a non-determined state for presence or absence of CHD in Ts1Cje. References are given in the text.

Restoring the disomy of the genes present in the *App-Runx1* fragment in the Ts65Dn model (double transgenic Ts65Dn/Ms5Yah mice) resulted in the rescue of Ts65Dn postnatal lethality, indicating that one or more genes present on this region are responsible for the observed lethality and that Ts65Dn trisomic genes located on Mmu17 are not major players in this phenotype. Our finding, combined with the different observations of CHD and lethality present or absent from other DS models [3], [16] and summarized on Figure 7. This restricts the list of candidates genes for the cardiac defects to a few genes, namely *Sod1*, *Tiam1* and unknown predicted genes such as *Gm10789* or *Gm2771* that are trisomic in Dp16(2)Yey mice which display CHD but are only in two copies in the Ts1Cje, Dp(16)1Yey/Df(16)2Yey, and Ts65Dn/Ms5Yah models. *Sod1* was found decreased in Ms5Yah but not overexpressed in Ts65Dn. On the contrary *Tiam1* was found increased in Ts65Dn heart and not significantly downregulated in the Ms5Yah. Thus *Tiam1* is a candidate for cardiac defect in Ts65Dn. *Tiam1* encodes an ephrin related receptor that influences synapse functions and controls epithelial tight junctions [39]. It may contribute to Ts65Dn heart defects together with additional genes such as *Bach1* and *Rcan1* which were found deregulated in Ts65Dn and Ms5Yah arrays but not in Ts65Dn/Ms5Yah samples. Nevertheless in Ts65Dn/Ms5Yah mice the monosomy-induced lethality of the Ms5Yah allele is in part rescued but not as complete as for the Ts65Dn allele. Somehow some genes that are not included in the *Tiam1-Cbr* overlap between Ms5Yah and the *Df(16Tiam1-Kcnj6)Yey/+*, but located at the boundaries of the considered region, i.e. in the *Mrpl39-Tiam1* or *Kcnj6-Zfp295* intervals, must have a major effect on survival of the Ms5Yah mice. However, the CHD observed in Ts65Dn dead pups is lower than that observed in DS patients, indicating that one or more gene outside of the *Mrpl39-Zfp295* region are contributing to CHD.

Many aspects of the ECG phenotypes observed in adult Ts65Dn mice were rescued by re-establishing euploidy of the *App-Runx1* region in double transgenic Ts65Dn/Ms5Yah mice. Reduced heart rate and prolonged QRS and QT observed in Ts65Dn mice were all back to normal in Ts65Dn/Ms5Yah mice, whereas the first degree block (PR) was only partially restored. The essential role of the *App-Runx1* region in the appearance of the Ts65Dn electrocardiographic pattern is hence highlighted by the reduced ECG phenotype in Ts65Dn/Ms5Yah mice. The obvious changes recorded in the waves registered by precordial recording between Ts65Dn and Ts65Dn/Ms5Yah and the rescue of the QT phenotype point again to a major contribution of the *App-Runx1* region to Ts65Dn ECG phenotypes. However, ECG defects are not completely recovered in the Ts65Dn/Ms5Yah compound animals and are probably induced by complex interactions between genes located in distinct regions of the Mmu16.

Recent evidence suggests that the same genes that cause defects in heart development and CHD might be involved in cardiac dysfunction such as abnormal electrical conduction and diminished contractile function [40]. Recent studies on adult DS individuals free of any CHD point to cardiovascular dysfunctions such as altered heart rate regulation [7], [8], valvular dysfunction [9], [10], bradycardia and AV block [11], [12]. Some of these life-threatening pathologies such as the observed bradycardia in Ts65Dn and altered heart rate control are also observed in another DS mouse model overexpressing *Kcnj6* [41]. Thus post-natal lethality in Ts65Dn or Ms5Yah does not necessarily involve overt CHD but could be related to altered cardiovascular function. Likely targets are the Na and K currents and/or Cx deficiency. In this respect, calcineurin-NFAT signaling controlled by *Rcan1* is clearly involved in the formation of the *annulus fibrosis* between the auricles and ventricles as well as in the formation of the valvules [40], [42], [43]. Post-natal lethality in DS hence might be the result of a complex set of different events with the *App-Runx1* region that triggers conduction defects and contributes to the risk of CHD.

We compared transcriptional profiles of RNA from adult mouse heart of 2n, Ts65Dn, Ms5Yah and Ts65Dn/Ms5Yah mice in order to determine aneuploid genes that are sensitive to gene dosage and hence might be candidate for the heart phenotypes, and to attempt to correlate observed transcript level differences to pathways impacted by the different aneuploidies. Most of the expressed triplicated/monosomic genes were up-regulated in Ts65Dn, down-regulated in Ms5Yah and expressed at similar levels in wt and in Ts65Dn/Ms5Yah hearts. 73 genes located on the Ts65Dn chromosome were overexpressed in the heart with a ratio versus wt of 1.34±0.15; only 12 were not found deregulated. 19 of those genes were located on the Mmu17 region triplicated in the Ts65Dn [20]. In the Ms5Yah heart, 30 genes from the *App-Runx1* interval whose expression was detected in the heart were down-regulated to 0.68±0.08 expression level compared to wt and 3 genes, *Tiam1*, *Slc5a3* and *Mrps6*, were not affected by decrease in copy number. For most of those aneuploid genes, expression level returned to normal (1.10±0.1) in Ts65Dn/Ms5Yah double transgenic mice. The overall difference in gene expression levels between the different mouse models can be explained by gene dosage. Our data support the hypothesis that a triplicated Hsa21 causes a 50% increase in trisomic genes expression as a primary dosage effect [44]–[47]. Conti and colleagues showed that the mean ratio between trisomic and euploid genes was 1.58 for Hsa21 genes and close to 1 for the genes on other chromosomes [48], [49]. Nevertheless we found some interesting exception: *Atp6j* expression is not affected by gene copy number while *Tiam1* is more sensitive to increase copy number. In Ts65Dn heart, trisomic genes *Sod1*, *Atp5o*, *Hcls, Ripply3, Psmg1*, and *Sh3bgr* were not affected by the trisomy while *Slc5a3* and *Mrps6*, back to two copies in Ts65Dn/Ms5Yah mice, were still overexpressed.

Detailed expression analysis highlighted a list of 151 deregulated genes that were categorized in 7 groups (Table 6, Table S1). 82 of the 151 genes were located outside the aneuploid regions and on diverse chromosomes. In the first group of genes, 39 genes were up- and 3 down-regulated specifically in Ts65Dn heart (no deregulation in Ms5Yah hearts) and their deregulation was a consequence of trisomic genes located outside of the *App-Runx1* interval. 13 genes were found on the proximal part of the Mmu17 of the Ts65Dn chromosome, 14 trisomic from the Mmu16 and 12 located elsewhere in the genome. With the second group we identified 33 genes of which 26 from the *App-Runx1* interval, that were deregulated in both Ts65Dn and Ms5Yah and compensated in Ts65Dn/Ms5Yah double mutant mice. Group 3 encompassed 16 genes specifically deregulated by in Ts65dn hearts while 40 genes specific of the Ms5Yah heart are found in group 4. We also identified 3 additional groups (5, 6 and 7) containing 10, 10 and 7 genes respectively, with expression level affected by combination of aneuploidies. For example group 5 were specific for the Ms5Yah with similar level in the Ts65Dn/Ms5Yah mice. Interestingly genes from the seven groups contribute to pathways related to the observed phenotypes. 25 genes out of 151 are associated with embryonic lethality, growth defect, and premature death (Mouse Genome Database (MGD) November 2011, [50]). We found 9 genes from group 1 (*Dyrk1a, F5, Gabpa, Hmgn1, Pde10a, Morc3, Slc5a3, Tfb1m* and *Vwf*) that could be involved in the Ts65Dn perinatal lethality and 9 from group 2 (*App, Ifnar1, Itsn1, Ltn1, N6amt1, Slc8a1/Ncx1, Synj1, Usp16* and *Rcan1*), for which mutation impaired embryonic viability and growth that could contribute to Ts65dn and to the Ms5Yah impaired viability. In addition *Amot, Ccne2*, *Cerk, C1qa* and *Fzd3* from group 4, could contribute to growth retardation and premature death [51], [52]. Other genes from groups 5 and 7 such as the *Grin2b* or *Psmg1* can still contribute to the perinatal lethality observed in the Ts65Dn/Ms5Yah [53], [54]. Changes in the expression of this series of genes might explain the birth defects observed in the Ts65Dn or the Ms5Yah mice. Only 6 genes out of 151 were found associated with heart dysfunction. More specifically, *Tfb1m* (group 1) causes abnormal heart development and physiology that could affect the viability of the Ts65Dn/Ms5Yah mice [55]. *Adam19, Slc8a1/Ncx1* and *Rcan1* from group 2 are able to induce various types of heart defects from irregular heartbeat to ASD and VSD [56]–[59]. In addition *Ripply3* and *Ccne2* (group 4) loss-of-functions potentially induce VSD [60], [61]. All these data show that the *App-Runx1* region play an important role in the heart defects and lethality observed in Ts65Dn and suggest some pathways altered in DS heart. Overall the analysis reveals the complexity of the phenotype with several trisomic genes along the Hsa21 working alone or in cooperation to contribute to the whole range of heart defects observed DS. Whole genome expression analysis pointed at some deregulated genes, whose contribution should be further analyzed. These data were obtained from adult trisomic mice, and thus do not give information about expression at the embryonic or postnatal states. We believe that further molecular and electrophysiological studies at postnatal states could thus give important information about genes involved in early postnatal lethality and should confirm or point to new candidate genes.

## Materials and Methods

### Ethics statement

Mice were handled with the agreement of the local ethical committee and in accordance with the European Council Guidelines for the Care and Use of Laboratory animals (accreditation 7320). They were housed under a 12 h/12 h light-dark cycle in TAAM-CNRS husbandry at Orléans (France) (certificate C45-234-6) and fed on a standard rodent chow. YH, as the principal investigator in this study, was granted the accreditation 45-31 and 67–369 to perform the reported experiments.

### Mouse lines

Female B6EiC3Sn a/A-Ts(17^16^)65Dn (Ts65Dn) mice were purchased from the Jackson Laboratory (Bar Harbor, ME). They were mated with F1 B6C3B males, in which the B6 are C57BL/6J mice and C3B are sighted C3H/HeH, a congenic line for the BALB/c allele at the *Pde6b* gene [62], to establish a breeding colony as described by Braudeau *et al.* [63]. Mice bearing the deletion between the *App* and *Runx1* genes were obtained by *in vivo* TAMERE [64], [65]. Briefly, a loxP site was introduced by homologous recombination in embryonic stem cells at the *App* locus using MICER vector [66] and the corresponding *App^tm1Yah^* mouse line was generated. *Runx1^tm1Yg^* mice were described previously [67]. Mice containing the two loxP sites were mated with the mouse line *Tg(Pgk1-Cre)1Lni*, expressing the Cre recombinase under the control of the early acting phosphoglycerate kinase-1 promoter [68]. Females that inherited both the Cre transgene and the two loxP sites on the same chromosome (cis configuration) were mated with wild-type B6 males. In oocytes of these females, Cre expression starting at the diploid phase induces loxP sites recombination leading to the deletion of the *App*-*Runx1* region in females gametes and to an offspring carrying the Del(16*App-Runx1*)5Yah noted here Ms5Yah, monosomic for this region (Figure S1). All the lines were kept on an F1 B6C3B background as described previously [20], [63]. After combining the Ms5Yah and Ts65Dn models, animals carrying both the Ts65Dn allele and the Ms5Yah allele were referred to as Ts65Dn/Ms5Yah and those neither trisomic nor monosomic were used as wt control individuals.

### Mouse genotyping

For identification of both Ms5Yah and Ts65Dn alleles, genomic DNA was isolated from tail biopsies using the NaCl precipitation technique. The Ts65Dn allele was identified using a Taqman qPCR protocol with differential analysis of quantity between *Apob* (housekeeper gene) and *Mx1* (gene of interest). *Apob* forward (CACGTGGGCTCCAGCATT), *Apob* reverse (TCACCAGTCATTTCTGCCTTTG), *Mx1* forward (TCTCCGATTAACCAGGCTAGCTAT) and *Mx1* reverse (GACATAAGGTTAGCAGCTAAAGGATCA) primers were purchased from SIGMA-Aldrich, and Taqman MGB probes *Mx1* FAM (6-FAM-CCTGGTCGCTGTGCA-MGB-NFQ) and *Apob* HEX (HEX-CCAATGGTCGGGCAC-MGB-NFQ) from Applied Biosystem. PCR conditions were as follows: (1) 50°C for 2 min, (2) 95°C for 10 min, (3) 95°C for 15 sec, (4) 60°C for 1 min (steps 3 and 4 were repeated 50 times). Alternatively we used the PCR–based protocol [20].

The Ms5Yah allele was identified by PCR using one Fwd primer (5′-ATCCGGGAATGGTCCCTA-3′) specific for the wt allele, one Fwd primer (5′-CAAGCACTGGCTATGCATGT-3′) specific for the Ms5Yah allele and a Ms5Yah/wt Rev (5′-GTTCGTTGCCTGAAGGAGAG-3′) primer common to both alleles. PCR reactions gave wt and Ms5Yah products of 482 bp and 328 bp long respectively.

### Gross morphology and histology

Breeding cages were checked twice every day and dead pups were removed, fixed in 4% paraformaldehyde in phosphate-buffered saline (PBS) for 24 hours and rinsed in PBS. Micro-dissections were realized to assess cardiovascular malformations using a Leica MZFL-III dissecting microscope equipped with a Leica DC200 digital camera. Aortic arches were then removed and hearts embedded in paraffin using a Leica TP 1020 tissue processor. Serial sections between 5 µm and 8 µm were fixed on Stick-On coated slides (LABOnord). Slides were dewaxed in xylene, rehydrated and stained in hematoxylin and 0.2% eosin. Images were captured using a Leica M420 brightfield macroscope equipped with a Photometrics Cool Snap digital camera.

### Electrocardiogram recording and analysis

Electrocardiogram (ECG) was recorded under urethane anesthesia (1.33 g/kg i.p.; Sigma) as previously described [41]. Adult mice (4 to 9 months old) were placed in a supine position in a Faraday cage. Ambient air was maintained at 25–26°C, close to the neutral temperature. Three leads consisting of 50 µm thin Ag-AgCl wires were placed subcutaneously in the forelegs and the left hind leg. Four precordial derivations were obtained by placing two leads on the sternal plate at its end (xiphoid cartilage, Vs), and midway at the junction with the fourth rib pair (Vms) and the two other leads on right (V1) and left (V4) sides of the sternum forming a square with Vs and Vms. V1 and V4 aproximatively mimicked human V1 and V4 leads. A tiny incision of the epidermis was necessary to ensure a good electrical contact. Each derivation recording was amplified (10^3^) with a 1–500 Hz bandwidth (IsoDAM8 amplifiers; WPI, Aston, UK) and continuously monitored on a Gould oscilloscope (20 MHz, type 1421). Acquisition was routinely performed at 2 kHz (IOX software v. 1.582; EMKA Technologies, Paris, France). R, S, and RS wave amplitudes and heart rate (HR) were averaged and monitored on line every 10 s. After 1 h of control recording, the 6 peripheral and 4 precordial leads were recorded at 5 kHz for one minute each.

HR mode was obtained from HR averaging over 10 s. Off-line analysis of ECG was performed with ECG-auto software (v. 1.5.11 EMKA Technologies). The ECG wave analysis used ECG libraries made up from original tracings. Measurements were performed every 20 sec, and reported values are the average of five measurements made on individual wave complexes taken over the first seconds of each interval. As the mouse ECG displayed no real electrical zero, this was taken as the average of five consecutive points taken between the end of P wave and the beginning of Q (or R) wave (from time 15 ms before Q/R wave beginning) as in infants. We followed Liu *et al.* to label the second wave adjoining the QRS complex as a J wave [69]. The delayed T wave was constantly identified on the Vms lead that was used for QT measurement. Corrected QT for RR interval changes (QTc) was calculated as previously described: QTc = QT/(RR/100)^1/2^. Electrical axis was determined from the algebraic sum of the QRS amplitude in aVF and DI. Flecainide (Sigma; 20 mg/kg) was prepared in distilled water and injected intraperitoneally.

Results are given as mean values ± standard error of the mean (sem). Statistical tests were performed with Sigma Stat software (Systat Software). We used the Student's t-test and ANOVA followed by the Student-Newman-Keuls (SNK) multiple-comparison tests. Proportions were analyzed with the Fisher exact test. Significance was set at *P*<0.05.

### Total RNA extraction

For Affymetrix arrays and qPCR validation, hearts were isolated from Ts65Dn, Ms5Yah, Ts65Dn/Ms5Yah and wt control mice (N = 5 per group) at 5 months of age and flash frozen. For cardiac connexins and sodium channels expression, hearts were isolated from 7 Ts65Dn and 5 wt adult mice at 4 months of age, and ventricles were separated from atria and flash frozen. Total RNA was prepared using Trizol (Invitrogen) according to the manufacturer's instructions. Samples quality was checked using an Agilent 2100 Bioanalyzer (Agilent Technologies).

### Whole-genome expression arrays

Biotinylated cDNAs were prepared from total RNAs and hybridized onto GeneChip Mouse GENE 1.0ST arrays (Affymetrix). Chips were washed and scanned on the Affymetrix Complete GeneChip instrument system generating digitized image data files. Raw data was processed with the Robust Multiarray Average (RMA) algorithm developed by Irizarry *et al.* [70] and values log transformed using Partek (Partek Inc.) and GeneSpring (Agilent Technologies) software. Statistical analysis was performed using GeneSpring (one way ANOVA) and the 213 genes with a p-value *P*<0.1 were selected for clustering analysis. Hierarchical clustering was carried out with Cluster3.0 software using Euclidian distances to calculate the distances between the genes and between the samples. Calculated distances were then clustered by complete linkage clustering. Post-Hoc analysis using GeneSpring gave a list of statistically deregulated genes. Known mammalian phenotypes database (Mouse Genome informatics, Jackson Laboratory) and functional annotation clustering using Database for Annotation, Visualization and Integrated Discovery (DAVID) bioinformatics were performed to estimate the potential impact of deregulated genes in transgenic mice. This latter tool mainly provides typical batch annotation and gene-GO term enrichment analysis to highlight the most relevant Gene Ontology (GO) terms associated with a given genes list [71], [72]. The microarray data were submitted to the Array Express Home under the accession number E-MEXP-3355.

### qPCR analysis

After DNase treatment with TurboDNA-free (Applied Biosystems), 1 µg of total RNA was converted to cDNA using Superscript III (Invitrogen) primed with poly d(T) and random hexamers. *Ppia*, *Gnas*, *Hprt1*, and *Pgk1* were selected as reference genes for normalization for both Affymetrix array validation and cardiac connexins and sodium channels gene expression experiments. Primers and Taqman probes were designed using Primer3 software (Table S2). Taqman 5′FAM-3′BHQ1 probes were purchased from Eurogentec (Angers, FR). All reactions used FastBlue qPCR Mastermix from Eurogentec (Angers, FR). All PCRs were performed in triplicate and ran in a Mastercycler epRealplex (Eppendorf) with the following conditions : 50°C for 2 min, 95°C for 5 min and 40 cycles of 95°C 15 s/60°C for 1 min. Raw Cycle threshold (Ct) were obtained using Realplex software (Eppendorf). Values with a deviation over 0.3 Ct with respect to the median were considered outliers and excluded. Selection of normalization genes and normalization factor were determined using GeNorm software [73]. To assess the difference in gene expression between Ts65Dn and wt samples we performed Student t-test. Significance was set at *P*<0.05.

## Supporting Information

Figure S1 Ms5Yah mouse model creation and validation. *App-Runx1* region (A) on Mmu16 was targeted for in vivo Cre/loxP recombination by inserting a loxP site on *App* locus in *Runx1^tm1Yg^* (B). Recombination using Tg(Pgk1-*cre*)1Lni led to deletion of the floxed fragment creating Ms5Yah mouse model. Chimeras and monosomic mice were distinguished using Southern Blot genotyping (C) and the deletion was confirmed by CGH arrays (D).
(TIF)

Table S1 List of deregulated genes in Affymetrix hybridization arrays. This list was obtained by using ANOVA with Bonferroni correction and fixing a threshold of *P*<0.1. Genes in pink. orange and blue are genes located respectively within the centromere-*Pde10a* region on Mmu17. the *Mrpl39-Runx1* and *Runx1-Zfp295* region on Mmu16 trisomic in Ts65Dn mice. We calculated the mean, the standard error to the mean for the expression ratio and the Student-t-test comparing mutant versus wt and annotated with * if *P*<0.05. ** if *P*<0.01 and *** if *P*<0.001. Overexpressed genes with an expression ratio>1.2 are underlined in red whereas down-regulated gene with a ratio<0.8 are in green. Normal expression ration between 0.8 and 1.2 are underlined in grey.
(PDF)

Table S2 Sequences and Tm of primers and Taqman probes used for qPCR analyses. Genes in bold were used as housekeepers.
(PDF)
