# Supplementary figures and images for: The App-Runx1 Region Is Critical for Birth Defects and Electrocardiographic Dysfunctions Observed in a Down Syndrome Mouse Model
Source: PLoS Genet. 2012 May 31;8(5):e1002724. doi: 10.1371/journal.pgen.1002724 (PMC3364940; doi:10.1371/journal.pgen.1002724)

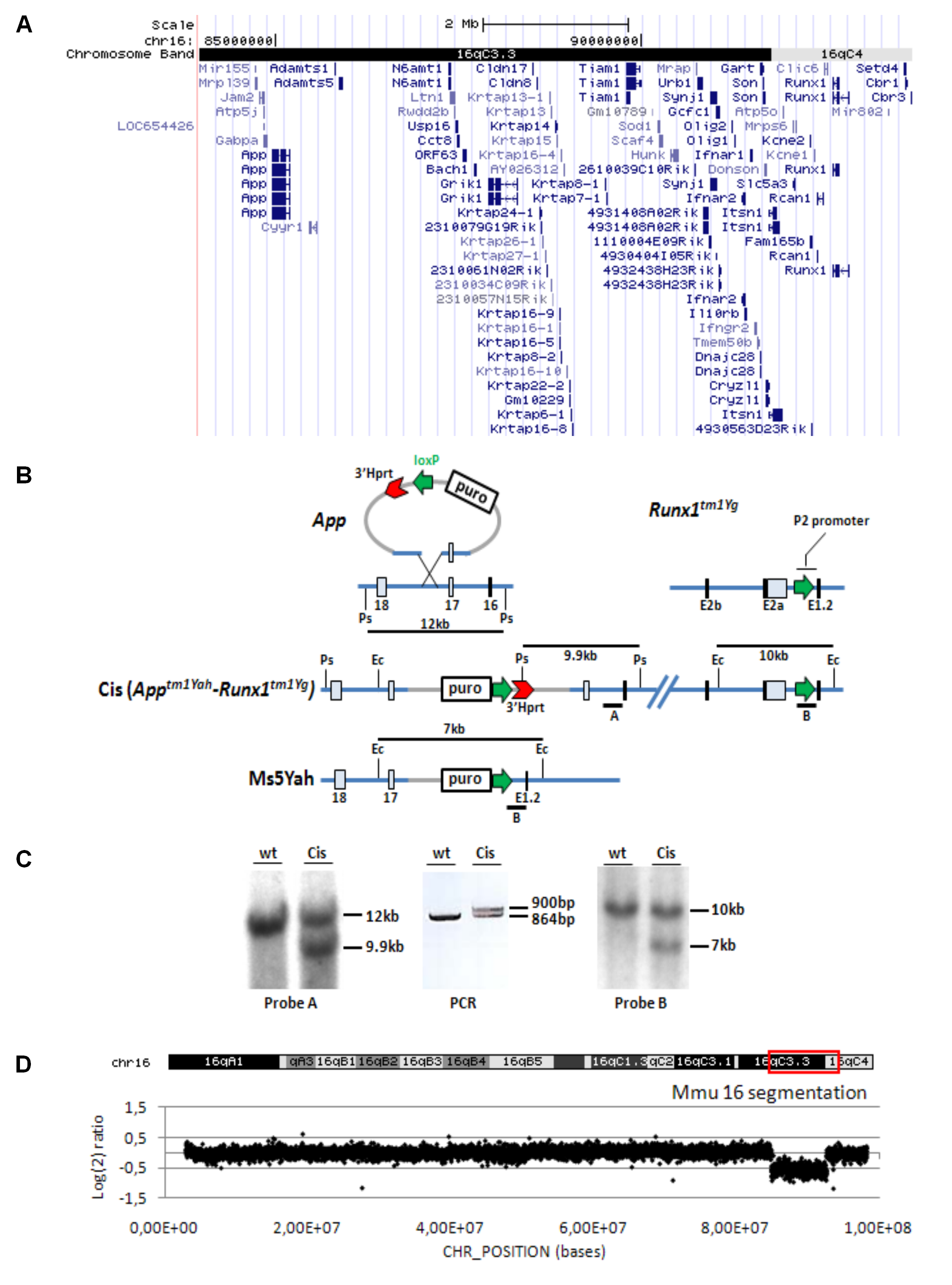

Supplement: Figure S1 — Ms5Yah mouse model creation and validation. App-Runx1 region (A) on Mmu16 was targeted for in vivo Cre/loxP recombination by inserting a loxP site on App locus in Runx1tm1Yg (B). Recombination using Tg(Pgk1-cre)1Lni led to deletion of the floxed fragment creating Ms5Yah mouse model. Chimeras and monosomic mice were distinguished using Southern Blot genotyping (C) and the deletion was confirmed by CGH arrays (D). (TIF) [file pgen.1002724.s001.tif]
