# Supplementary material for: The App-Runx1 Region Is Critical for Birth Defects and Electrocardiographic Dysfunctions Observed in a Down Syndrome Mouse Model
Source: PLoS Genet. 2012 May 31;8(5):e1002724. doi: 10.1371/journal.pgen.1002724 (PMC3364940; doi:10.1371/journal.pgen.1002724)
Supplement: Table S1 — List of deregulated genes in Affymetrix hybridization arrays. This list was obtained by using ANOVA with Bonferroni correction and fixing a threshold of P<0.1. Genes in pink. orange and blue are genes located respectively within the centromere-Pde10a region on Mmu17. the Mrpl39-Runx1 and Runx1-Zfp295 region on Mmu16 trisomic in Ts65Dn mice. We calculated the mean, the standard error to the mean for the expression ratio and the Student-t-test comparing mutant versus wt and annotated with * if P<0.05. ** if P<0.01 and *** if P<0.001. Overexpressed genes with an expression ratio>1.2 are underlined in red whereas down-regulated gene with a ratio<0.8 are in green. Normal expression ration between 0.8 and 1.2 are underlined in grey. (PDF) [file pgen.1002724.s002.pdf]

			Genotype	wt			Ts65Dn				Ms5Yah				Ts65Dn/Ms5Yah			
**Group**	**Gene Name**	**Chr**	**Anova**	mean		sem	mean		sem	test post hoc	mean		sem	test post hoc	mean		sem	test post hoc
1	*Arid1b*	17T	***	1.03	±	0.02	1.3	±	0.05	***	1.02	±	0.03		1.31	±	0.02	***
1	*Gtf2h5*	17T	**	1.06	±	0.03	1.39	±	0.09	***	1.07	±	0.08		1.52	±	0.09	***
1	*Tfb1m*	17T	*	0.96	±	0.07	1.21	±	0.11	*	0.9	±	0.04		1.24	±	0.06	***
1	*Rnaset2a*	17T	***	0.95	±	0.02	1.23	±	0.04	***	0.98	±	0.02		1.26	±	0.02	***
1	*Snx9*	17T	***	1.04	±	0.04	1.4	±	0.05	***	1.07	±	0.04		1.38	±	0.03	***
1	*5730437N04I*	17T	***	0.91	±	0.04	1.41	±	0.11	***	0.85	±	0.04		1.44	±	0.07	***
1	*Tulp4*	17T	***	0.99	±	0.01	1.45	±	0.04	***	1.01	±	0.14		1.53	±	0.04	***
1	*Scaf8*	17T	***	1,00	±	0.03	1.34	±	0.07	***	0.95	±	0.02		1.27	±	0.03	***
1	*Tiam2*	17T	***	1,00	±	0.08	1.54	±	0.05	***	0.99	±	0.1		1.49	±	0.09	***
1	*Fgfr1op*	17T	***	0.91	±	0.06	1.38	±	0.07	***	0.89	±	0.02		1.29	±	0.06	***
1	*Brp44l*	17T	*	1.05	±	0.05	1.21	±	0.04	**	1.04	±	0.02		1.21	±	0.06	**
1	*Dynlt1*	17T		1.03	±	0.06	1.37	±	0.1	**	1.16	±	0.15		1.48	±	0.11	***
1	*Pde10a*	17T		0.94	±	0.08	1.24	±	0.1	**	0.95	±	0.1		1.21	±	0.06	**
1	*Mrpl39*	16T	***	0.99	±	0.02	1.29	±	0.06	***	1.01	±	0.05		1.32	±	0.06	***
1	*Jam2*	16T	*	1.06	±	0.05	1.37	±	0.15	**	1.07	±	0.02		1.4	±	0.08	***
1	*Gabpa*	16T	**	1,00	±	0.03	1.29	±	0.1	**	0.96	±	0.05		1.24	±	0.07	***
1	*Mrps6*	16T	**	1.08	±	0.04	1.64	±	0.14	***	0.83	±	0.14		1.21	±	0.03	**
1	*Slc5a3*	16T		1.11	±	0.14	1.7	±	0.18	**	0.84	±	0.2		1.22	±	0.13	
1	*Pigp*	16T	**	1.07	±	0.06	1.38	±	0.1	**	0.88	±	0.07		1.2	±	0.05	*
1	*Dyrk1a*	16T	***	0.99	±	0.02	1.29	±	0.03	***	0.97	±	0.02		1.26	±	0.03	***
1	*Cbr1*	16T	*	1.06	±	0.05	1.33	±	0.06	***	1.09	±	0.07		1.39	±	0.12	**
1	*Hmgn1*	16T	*	0.98	±	0.02	1.26	±	0.11	**	1.12	±	0.06		1.27	±	0.03	***
1	*Brwd1*	16T	**	1.01	±	0.08	1.33	±	0.08	***	0.98	±	0.04		1.31	±	0.05	***
1	*Dopey2*	16T	***	1.01	±	0.04	1.35	±	0.05	***	0.96	±	0.06		1.32	±	0.06	***
1	*Morc3*	16T	***	0.98	±	0.03	1.38	±	0.06	***	0.91	±	0.05		1.28	±	0.05	***
1	*Ttc3*	16T	***	0.98	±	0.03	1.41	±	0.07	***	0.94	±	0.06		1.34	±	0.05	***
1	*C2cd2*	16T	***	0.93	±	0.06	1.44	±	0.11	***	0.89	±	0.03		1.46	±	0.09	***
1	*AU021092*	16	**	1.02	±	0.08	1.26	±	0.11	*	0.87	±	0.04		1.32	±	0.1	**
1	*Heph*	X	***	1.09	±	0.08	1.27	±	0.06	*	1.11	±	0.05		1.43	±	0.1	**
1	*Fbln2*	6	**	1.03	±	0.08	1.3	±	0.08	**	0.96	±	0.03		1.34	±	0.08	***
1	*AI593442*	9	**	0.95	±	0.09	1.83	±	0.39	**	0.94	±	0.06		1.68	±	0.25	**
1	*Pdgfd*	9	*	1.03	±	0.09	1.23	±	0.02	**	0.94	±	0.04		1.21	±	0.08	*
1	*F5*	1		1.12	±	0.07	1.49	±	0.14	**	1.08	±	0.12		1.22	±	0.05	
1	*Vwf*	6		1.06	±	0.05	1.32	±	0.08	**	1.02	±	0.08		1.27	±	0.08	**
1	*Pcbd1*	10		1.14	±	0.08	1.66	±	0.22	**	0.86	±	0.1		1.34	±	0.3	
1	*P2ry12*	3		1.03	±	0.04	1.26	±	0.08	**	1.01	±	0.06		1.22	±	0.07	**
1	*Lrp2bp*	8		0.92	±	0.05	0.65	±	0.04	***	0.83	±	0.13		0.64	±	0.07	***
1	*Htr2a*	14		1.01	±	0.08	0.7	±	0.06	***	0.83	±	0.07		0.67	±	0.1	**
1	*Clk1*	7		0.91	±	0.07	0.77	±	0.05	*	0.85	±	0.05		0.72	±	0.03	**
2	*Tmem50b*	16T	**	0.93	±	0.05	1.5	±	0.14	***	0.63	±	0.14		1.18	±	0.09	
2	*Rwdd2b*	16T	*	1,00	±	0.05	1.25	±	0.12	*	0.74	±	0.11	*	1.03	±	0.09	
2	*N6amt1*	16T	**	1.04	±	0.1	1.27	±	0.1	*	0.67	±	0.11	**	0.97	±	0.07	
2	*Il10rb*	16T	*	1.02	±	0.04	1.31	±	0.1	**	0.75	±	0.12	**	1.04	±	0.07	
2	*Bach1*	16T	**	1.01	±	0.02	1.3	±	0.1	***	0.75	±	0.1	**	1.03	±	0.06	
2	*Ltn1*	16T	***	1.01	±	0.07	1.34	±	0.09	**	0.62	±	0.12	**	0.91	±	0.06	
2	*Ifnar1*	16T	***	0.92	±	0.04	1.22	±	0.05	***	0.61	±	0.1	**	0.93	±	0.06	
2	*Fam165b*	16T	*	1,00	±	0.03	1.32	±	0.07	***	0.77	±	0.16	*	1.08	±	0.08	
2	*Cyyr1*	16T	**	0.96	±	0.05	1.28	±	0.1	**	0.59	±	0.13	**	0.93	±	0.04	
2	*Synj1*	16T	***	1.05	±	0.02	1.4	±	0.06	***	0.66	±	0.11	**	1.02	±	0.04	
2	*Scaf4*	16T	***	0.99	±	0.01	1.33	±	0.05	***	0.68	±	0.11	**	0.95	±	0.02	
2	*Itsn1*	16T	***	0.96	±	0.03	1.29	±	0.04	***	0.65	±	0.1	*	1,00	±	0.05	
2	*Cryzl1*	16T	**	1.05	±	0.03	1.42	±	0.14	**	0.71	±	0.12	**	1,00	±	0.06	
2	*Gart*	16T	**	0.94	±	0.03	1.3	±	0.06	***	0.67	±	0.11	**	0.91	±	0.03	
2	*Urb1*	16T	***	0.92	±	0.03	1.31	±	0.08	***	0.64	±	0.09	**	0.88	±	0.04	
2	*Ifnar2*	16T	**	0.99	±	0.03	1.42	±	0.12	***	0.59	±	0.14	**	1.02	±	0.07	

2	*Usp16*	16T	**	1,00	±	0.06	1.47	±	0.14	***	0.64	±	0.14	**	1,00	±	0.06
2	*Cct8*	16T	***	0.91	±	0.05	1.33	±	0.06	***	0.51	±	0.12	**	0.89	±	0.03
2	*Son*	16T	***	0.97	±	0.03	1.4	±	0.05	***	0.58	±	0.12	**	0.95	±	0.05
2	*App*	16T	**	1.04	±	0.09	1.54	±	0.04	***	0.64	±	0.15	*	1.1	±	0.03
2	*Mrap*	16T	*	0.96	±	0.02	1.49	±	0.21	**	0.78	±	0.15		1.1	±	0.08
2	*Gcfc1*	16T	**	1.05	±	0.04	1.47	±	0.08	***	0.69	±	0.15	**	0.97	±	0.05
2	*Rcan1*	16T		1.04	±	0.14	1.57	±	0.14	**	0.8	±	0.17		1.13	±	0.11
2	*Usp16*	16T	**	1,00	±	0.06	1.47	±	0.14	***	0.64	±	0.14	**	1,00	±	0.06
2	*Dnajc28*	16T		0.91	±	0.04	1.23	±	0.16	*	0.59	±	0.18	*	0.86	±	0.05
2	*Son*	16T	***	0.97	±	0.03	1.4	±	0.05	***	0.58	±	0.12	**	0.95	±	0.05
2	*Mx2*	16T		0.94	±	0.07	1.26	±	0.35		0.64	±	0.05		1.05	±	0.08
2	*Irf7*	7		0.92	±	0.1	1.37	±	0.26	*	0.67	±	0.13		0.93	±	0.13
2	*Zfp583*	7		0.93	±	0.1	1.29	±	0.1	**	0.72	±	0.15		0.99	±	0.05
2	*Slc38a4*	15	**	1.14	±	0.09	0.76	±	0.06	***	1.27	±	0.14		0.86	±	0.07
2	*Slc8a1*	17		0.99	±	0.16	0.8	±	0.07		1.34	±	0.14		1.03	±	0.11
2	*Adam19*	11	*	1.1	±	0.09	1.28	±	0.2		1.89	±	0.34	*	1.06	±	0.09
2	*Vmn2r86*	10	*	0.96	±	0.08	1.59	±	0.22	**	1.38	±	0.13	**	1.1	±	0.12
3	*Serac1*	17T	**	0.95	±	0.06	1.23	±	0.06	***	0.88	±	0.05		1.2	±	0.06
3	*Tagap1*	17T	**	0.87	±	0.06	1.28	±	0.08	***	0.85	±	0.07		1.14	±	0.04
3	*Rps6ka2*	17T	**	0.93	±	0.08	1.33	±	0.11	**	0.83	±	0.07		1.15	±	0.08
3	*Tiam1*	16T	*	1.07	±	0.08	1.42	±	0.11	**	0.84	±	0.11		1.05	±	0.04
3	*Wrb*	16T	***	0.86	±	0.06	1.22	±	0.02	***	0.89	±	0.02		1.18	±	0.04
3	*St8sia5*	18		0.95	±	0.03	1.27	±	0.14	**	1.19	±	0.08		1.18	±	0.06
3	*Slc39a6*	18		0.99	±	0.03	1.25	±	0.11	**	0.97	±	0.04		1.14	±	0.08
3	*Abi3bp*	16		1.14	±	0.06	1.42	±	0.11	**	1.03	±	0.1		1.2	±	0.06
3	*BC055004*	5		0.97	±	0.07	1.5	±	0.22	**	0.84	±	0.07		1.14	±	0.23
3	*Dyrk1b*	7	*	0.95	±	0.04	0.73	±	0.04	***	0.82	±	0.02		0.84	±	0.03
3	*Ypel2*	11	*	0.88	±	0.05	0.79	±	0.06		1.18	±	0.1		0.9	±	0.1
3	*Chrna2*	14		1.09	±	0.05	0.78	±	0.1	**	0.8	±	0.04		0.81	±	0.09
3	*Ldhd*	8		0.97	±	0.06	0.79	±	0.05	**	0.93	±	0.03		0.86	±	0.03
3	*Gm16493*	9		0.9	±	0.07	0.71	±	0.07	*	1.01	±	0.07		0.89	±	0.06
3	*Pm20d1*	1		1.02	±	0.09	0.65	±	0.08	***	0.96	±	0.1		0.88	±	0.12
3	*Nudt8*	19	*	0.93	±	0.05	0.79	±	0.05	**	1.05	±	0.04		0.94	±	0.01
4	*Sft2d1*	17T	**	0.85	±	0.09	1.2	±	0.03		0.78	±	0.05		1.12	±	0.05
4	*Zdhhc14*	17T	**	0.85	±	0.06	1.11	±	0.12		0.74	±	0.02		1.1	±	0.07
4	*Ifngr2*	16T	**	0.92	±	0.03	1.13	±	0.08		0.65	±	0.09	*	0.96	±	0.05
4	*1110004E09R*	16T	**	0.93	±	0.05	1.2	±	0.09		0.63	±	0.11	**	0.87	±	0.03
4	*2610039C10R*	16T	*	0.9	±	0.08	1.09	±	0.16		0.61	±	0.06	**	0.86	±	0.08
4	*Sod1*	16T	*	0.97	±	0.03	1.16	±	0.05		0.76	±	0.09	**	0.96	±	0.03
4	*Atp5o*	16T	**	0.94	±	0.04	1.13	±	0.09		0.65	±	0.08	**	0.94	±	0.01
4	*Ripply3*	16T	**	0.91	±	0.06	1.15	±	0.09		0.77	±	0.06		1.1	±	0.06
4	*Sh3bgr*	16T		0.89	±	0.08	1.18	±	0.16		0.79	±	0.05		1.17	±	0.06
4	*Carkd*	8	*	0.94	±	0.04	0.93	±	0.04		0.79	±	0.01	**	0.91	±	0.05
4	*Amot*	X	*	1.02	±	0.06	1.02	±	0.12		0.72	±	0.06	*	1.01	±	0.02
4	*Pnck*	X	*	0.95	±	0.05	0.94	±	0.09		0.64	±	0.09	**	0.85	±	0.02
4	*Gm8566*	6	*	1,00	±	0.05	1.13	±	0.05		0.74	±	0.1	**	0.96	±	0.01
4	*Gm5436*	12	**	0.92	±	0.04	1.14	±	0.1		0.71	±	0.09	*	0.97	±	0.03
4	*Hmcn2*	2	*	0.92	±	0.07	1.12	±	0.13		0.78	±	0.06		1.19	±	0.11
4	*Scn4b*	9	**	0.9	±	0.15	1.06	±	0.22		0.34	±	0.08	*	1.12	±	0.18
4	*Rasl10b*	11	*	0.88	±	0.05	0.9	±	0.05		0.72	±	0.06		1.02	±	0.07
4	*Tnxb*	17		0.98	±	0.05	0.96	±	0.1		0.75	±	0.05		0.96	±	0.05
4	*Pde7b*	10		1,00	±	0.04	0.87	±	0.07		0.73	±	0.03	*	0.9	±	0.08
4	*C1qa*	4		0.86	±	0.06	1.01	±	0.08		0.74	±	0.07		1.07	±	0.06
4	*Ccne2*	4		0.97	±	0.05	0.99	±	0.07		0.71	±	0.06	*	0.82	±	0.07
4	*Crnkl1*	2		0.93	±	0.05	0.92	±	0.04		0.79	±	0.01		0.85	±	0.04
4	*Cerk*	15	*	1.05	±	0.05	1.11	±	0.1		1.29	±	0.06		0.94	±	0.03
4	*2810410L24R*	11		0.92	±	0.08	0.94	±	0.07		1.24	±	0.11		1.14	±	0.1
4	*Gm10400*	6		0.98	±	0.06	0.86	±	0.09		1.28	±	0.09		0.91	±	0.1

4	*2210021J22R*	15		1.06	±	0.06	1.05	±	0.07		1.35	±	0.02		1.08	±	0.08	
4	*Fam189a1*	7		0.98	±	0.02	1.01	±	0.07		1.23	±	0.08		1.15	±	0.05	
4	*Adamts6*	13		1.05	±	0.06	1,00	±	0.06		1.36	±	0.18		1.01	±	0.08	
4	*Zc3h12c*	9		1.09	±	0.04	1.09	±	0.06		1.3	±	0.03		1.08	±	0.06	
4	*Lrrfip1*	1		1.04	±	0.04	1.02	±	0.06		1.25	±	0.03		1.05	±	0.05	
4	*Lce1m*	3		1.01	±	0.03	1.07	±	0.05		1.25	±	0.06		1.15	±	0.09	
4	*Fzd3*	14		1,00	±	0.04	1.1	±	0.07		1.21	±	0.09		0.93	±	0.06	
4	*Pnrc1*	4		1.15	±	0.1	1.02	±	0.04		1.33	±	0.05		1.07	±	0.05	
4	*Tnni1*	1	*	1.02	±	0.03	1.01	±	0.1		1.34	±	0.1	**	1.13	±	0.05	
4	*mIR697*	4	*	0.96	±	0.04	0.99	±	0.03		1.25	±	0.09	**	1.06	±	0.06	
4	*St3gal4*	9	**	0.91	±	0.05	1,00	±	0.05		1.27	±	0.03	***	1.15	±	0.08	
4	*Mapk4*	18	*	0.92	±	0.09	0.86	±	0.07		1.28	±	0.13	*	0.91	±	0.07	
4	*Ybx2*	11	*	0.94	±	0.04	0.82	±	0.09		1.23	±	0.13	*	0.96	±	0.02	
4	*Cass4*	2	*	0.99	±	0.04	1.02	±	0.05		1.25	±	0.09	**	1.16	±	0.03	
4	*Clec12b*	6	*	1.02	±	0.03	0.93	±	0.06		1.33	±	0.1	*	1.05	±	0.08	
5	*Ccl24*	5	***	1.15	±	0.06	0.99	±	0.14		2.13	±	0.38	**	1.31	±	0.11	
5	*ENSMUSG00(*	4	*	1.03	±	0.02	1,00	±	0.04		1.22	±	0.06	***	1.23	±	0.08	**
5	*Olfr234*	15	*	1.03	±	0.04	1.09	±	0.07		1.39	±	0.16	**	1.38	±	0.07	***
5	*Disp2*	2	*	1.02	±	0.06	0.95	±	0.07		1.33	±	0.12	**	1.26	±	0.07	**
5	*Mmp9*	2		1.1	±	0.08	0.99	±	0.11		1.78	±	0.25		1.53	±	0.34	
5	*Tnrc4*	3		1.05	±	0.08	1.04	±	0.05		1.29	±	0.06		1.28	±	0.12	*
5	*Loxhd1*	18		0.97	±	0.08	1.14	±	0.11		1.32	±	0.09		1.2	±	0.06	**
5	*Adamts18*	8		1.03	±	0.03	1.1	±	0.07		1.23	±	0.06		1.34	±	0.12	**
5	*mIR125a*	17		1,00	±	0.03	1.13	±	0.05		1.27	±	0.06		1.22	±	0.08	**
5	*Grin2b*	6	**	1.03	±	0.03	1.15	±	0.03		1.23	±	0.04	***	1.26	±	0.03	***
6	*Fndc1*	17T	*	0.94	±	0.1	1.37	±	0.11	**	0.79	±	0.1		1.39	±	0.19	*
6	*Adamts1*	16T	*	1.22	±	0.25	1.48	±	0.2		0.64	±	0.12	*	1.07	±	0.1	
6	*Dscr3*	16T	***	1.12	±	0.09	1.72	±	0.08	***	1.29	±	0.04	*	1.73	±	0.07	***
6	*Bace2*	16T	***	1.08	±	0.08	1.84	±	0.11	***	1.24	±	0.09		1.85	±	0.15	***
6	*Plvap*	8	*	1.12	±	0.05	1.59	±	0.17	**	1.34	±	0.03	***	1.42	±	0.1	**
6	*S100a11*	3		1.09	±	0.08	1.43	±	0.11	**	1.21	±	0.1		1.4	±	0.07	**
6	*Kctd15*	7		1.02	±	0.07	1.21	±	0.05	**	1.3	±	0.08		1.3	±	0.09	**
6	*Abcd3*	3		0.97	±	0.03	0.77	±	0.06	**	0.79	±	0.06	*	0.75	±	0.05	***
6	*Tubb1*	2		1.24	±	0.23	1.93	±	0.2	**	1.17	±	0.15		1.5	±	0.13	
6	*Ppbp*	5	*	1.61	±	0.28	5.27	±	1.51	**	1.75	±	0.38		3.45	±	0.75	**
7	*Psmg1*	16T	**	0.86	±	0.06	1.18	±	0.1	**	0.87	±	0.06		1.26	±	0.09	
7	*Irs3*	5	**	0.98	±	0.02	0.94	±	0.05		1.09	±	0.07		1.21	±	0.05	***
7	*Gon4l*	3		0.98	±	0.01	0.84	±	0.06		0.91	±	0.05		0.79	±	0.05	***
7	*Sesn3*	9		1,00	±	0.03	0.85	±	0.06		0.87	±	0.04		0.79	±	0.05	***
7	*Fam163a*	1		1,00	±	0.03	1.07	±	0.05		1,00	±	0.06		1.21	±	0.07	**
7	*Slco3a1*	7		0.99	±	0.04	1.03	±	0.04		1.14	±	0.03		1.2	±	0.09	**
7	*Aard*	15		0.98	±	0.03	1.07	±	0.04		1.12	±	0.04		1.2	±	0.05	***
0	*Atp5j*	16T	**	0.96	±	0.02	1.06	±	0.04		0.97	±	0.02		1.14	±	0.01	
0	*Hlcs*	16T	**	0.95	±	0.04	1.16	±	0.05		0.83	±	0.07		1.2	±	0.09	
