# Supplementary material for: The App-Runx1 Region Is Critical for Birth Defects and Electrocardiographic Dysfunctions Observed in a Down Syndrome Mouse Model
Source: PLoS Genet. 2012 May 31;8(5):e1002724. doi: 10.1371/journal.pgen.1002724 (PMC3364940; doi:10.1371/journal.pgen.1002724)
Supplement: Table S2 — Sequences and Tm of primers and Taqman probes used for qPCR analyses. Genes in bold were used as housekeepers. (PDF) [file pgen.1002724.s003.pdf]

Gene	Forward primer	Reverse primer	Probe	Tm probe	Tm fwd primer	Tm rev primer
**Ppia**	AGGGTTCCTCCTTTCACAGAATT	TGCCATTATGGCGTGTAAAGTC	TCCAGGATTCATGTGCCAGGGTGG	73.1	61.5	62.1
**Gnas**	AGAACATCCGCCGTGTCTTC	CCTTCTTAGAGCAGCTCGTATTGG	CGTGACATCATCCAGCGCATGCAT	73.2	62.6	62.7
**PGK1**	ATTCTGCTTGGACAATGGAGC	AGGCATGGGAACACCATCA	AACTCCGTTGTCCTTATGAGCCACCTGG	72.1	61.5	62.4
**HPRT1**	GCAAACTTTGCTTTCCCTGG	ACTTCGAGAGGTCCTTTTCACC	CAGCCCCAAAATGGTTAAGGTTGCAAG	71.2	62,0	61,0
Cx40	ACTCCAGGGAGGAGGAAAGG	AGGAAGCTCCAGTCACCCAT	GGCCTCGGTCTCCTACTCTTGGCAA	71.3	61.9	61.1
Cx43	TGCCGGCTTCACTTTCATTA	GACCTTGTCCAGCAGCTTCC	AGACATGGGTGACTGGAGCGCCTTG	73.3	61.7	62.3
Cx45	TGGGTAACAGGAGTTCTGGTGA	CCTCTAGCAGGCGAGTCAGG	GGCAAACCAATTCCACCACCATGAG	71.1	61.9	62.6
Cx30.2	GGTCGTCTCTGGCTGGTGAT	ACGAACTCCTCCTGCTCGTC	TGATCTTCCGCATCCTGGTGCTGG	73.3	62.6	61.9
Scn1b	ACCAGCGTCGTCAAGAAGAT	GGTCAACACCACAATGAGCA	AGGTGGTGGACAAGGCCAACAGAGA	71,0	59.9	60.6
Scn5a	GGAAGAGTCCAGCAAACAGC	GGCTTCAGAGGATGTGGTCT	AATCCCAAGTTGTGTCTGGTGGCCA	71.5	60,0	59.3
Scn10a	GCCTCATCTTCTGGCTCATC	AATGGGTTGCTTCTGGTGTC	ATCATGGGTGTGAACCTCTTCGCCG	72.8	59.9	60,0
Usp16	CCTTCGGTTTGGCTCTGTCTT	GCATGCTGCTCCTGAGAATCT	GCCACAGCCCTGATGGCCACATT	73.2	62.9	62,0
Cct8	GGCTAAACAAATCACATCATATGGA	CAAACGCTTCAGCAAACTTCTTAA	GCATACTGTTCAAGCCCAGGACACGTCT	71.7	61.2	62,0
Bach1	CTGAGACGGACACGGAAGGT	GGGCATTGAATGGCAGCTT	ACCTCACAGTCCTGCTCCCTGGCA	72.4	62.6	63.4
Dyrk1a	TAACAGTGGGAGAGCCAGGT	ACCTGGGGACTGTGTGTCTC	AGCATCGACACAGCGGTGGACACTT	72,0	59.7	60,0
Sh3bgr	CCCTTCCCAATGGAGATGTA	TGTCCTCTTTCTGTGCCTCA	CAGAGGGTGCTGCAGAGGGAACAGA	71.9	59.7	59.6
